# Barriers, facilitators and strategies for implementing on-site hospital solar power in low- and middle-income countries: a systematic review, global prioritisation survey and development of an implementation tool

**DOI:** 10.1136/bmjgh-2026-023926

**Published:** 2026-06-18

**Authors:** Cortland Linder, Riya Tellis, Adewale Adisa, Dhruva Ghosh, Atul Suroy, Deepak Singh, Nick Marshall, Oliver Penney, Parvez D Haque, J C Allen Ingabire, Antonio Ramos-De La Medina, Dmitri Nepogodiev, Aneel Bhangu, NIHR Group on Environmentally Sustainable Hospitals, Cortland Linder

**Affiliations:** 1 University of Birmingham, Birmingham, UK; 2 Obafemi Awolowo University Teaching Hospital Complex, Ife, Osun, Nigeria; 3 Christian Medical College, Ludhiana, India; 4 Emmanuel Hospital Association, New Delhi, India; 5 Independent Researcher, Bristol, UK; 6 Independent Researcher, Hereford, UK; 7 University of Rwanda, Kigali, Kigali City, Rwanda; 8 Hospital Español de Veracruz, Veracruz, Mexico; 9 Department of Applied Health Sciences, University of Birmingham, Birmingham, UK

**Keywords:** Health policy, Implementation Science, Interdisciplinary Research, Systematic review, Global Health

## Abstract

**Introduction:**

Healthcare facility power-outages in low- and middle-income countries (LMICs) are common and affect patient outcomes. Decentralised solar energy has the potential to reduce power-outages in healthcare settings, yet adoption in LMIC remains modest. This study aimed to identify and prioritise the barriers, facilitators and strategies of on-site healthcare facility solar power implementation.

**Methods:**

Methodology was conducted in three phases. In phase 1, a systematic review was completed to summarise barriers and facilitators to implementation of on-site solar power in LMIC healthcare facilities. Any qualitative or mixed-methods study relating to solar power installation from 2009 to 2024 in LMICs was eligible to be included. Barriers and facilitators were extracted from the identified studies and mapped to the Consolidated Framework for Implementation Research. In phase 2, the importance of these barrier and facilitator factors was contextually prioritised through an international survey of healthcare workers on a 5-point Likert scale. In phase 3, strategies were developed from these barrier and facilitator factors to create a solar implementation tool (SOLAR-IT).

**Results:**

In phase 1, out of a total of 3678 individual records, 28 studies were included in the systematic review, from which 19 barriers and 29 facilitators were identified. In phase 2, the global survey was completed by 260 participants from 47 LMICs. The barrier with the highest ranked importance was the upfront capital expenditure of solar panel installation, while the most important facilitator was to reduce the cost of solar panels through government intervention. In phase 3, 23 strategies were synthesised within four domains to create the SOLAR-IT.

**Conclusion:**

This study describes globally prioritised implementation factors and a strategy tool, which can enable teams to speed up implementation of on-site solar power in a time of extreme global need.

**WHAT IS ALREADY KNOWN ON THIS TOPIC**

**WHAT THIS STUDY ADDS**

- This study identifies the barriers and facilitators to on-site hospital solar power, prioritises these in a global survey of frontline hospital workers and develops the solar implementation tool (SOLAR-IT) to support implementation.

**HOW THIS STUDY MIGHT AFFECT RESEARCH, PRACTICE OR POLICY**

- This study provides globally prioritised implementation factors and a new strategy tool to enable healthcare facilities to deploy solar power.
- Further implementation research is needed to further understand decentralised healthcare electricity programme delivery.

## Introduction

 Electricity is critical for delivery of essential medical services, lighting, refrigeration, medical devices such as ventilators, autoclaves and radiological investigations.^1^ However, an estimated one billion people in low- and middle-income countries (LMIC) are served by healthcare facilities with unreliable or absent electricity, and up to half of healthcare facilities in sub-Saharan Africa do not have reliable electricity.^2^ Facilities that are connected to the main electrical grid often suffer from frequent power-outages, occurring between 50 and 4600 hours per year in LMICs.^3^

Power outages or surges increase the risk of equipment failure, vaccine spoilage and disrupt procedures and access to 24-hour emergency care.^4^ Without electricity, healthcare facilities suffer procedure delays, reduced service, broken or unsterile equipment and inability to perform basic investigations or procedures.^5^ Hospitals without electricity are unable to remain open overnight, have poor community confidence and challenging working conditions for staff.^6^ Improving power improves maternity care and utilisation of health services.^7 8^ Many hospitals mitigate grid power-outages with on-site diesel generators. Together with the fossil fuels burnt to supply the electrical grid, this contributes to global warming. Overall, healthcare produces 4% of global carbon emissions, causing climate change, a health and climate emergency.^9^

There is global consensus on the urgent need to improve and decarbonise healthcare electricity, aligning with the United Nations Sustainable Development Goals 3 (health), 7 (affordable and clean energy) and 13 (climate action).^2 10^ One viable solution is harnessing solar energy using on-site healthcare facility solar power. Solar power can either be implemented as a stand-alone off-grid power system or integrated into conventional electricity grids or generators to provide hybridised power.^11^ Although the upfront capital costs of installing solar power remain high, these are rapidly decreasing; the average cost of solar panel modules has reduced by about 93% over the last decade.^2 12^ Additionally, solar power systems have substantially lower operational costs than diesel generators or the electrical grid, providing a long-term financial advantage for the hospital.^13–22^

Despite evidence showing financial, environmental and structural benefits of implementing on-site solar power, investment and uptake around the world has been limited.^23^ Implementation of on-site solar power is complex, requiring high upfront costs, skilled engineers and robust maintenance plans to ensure longevity.^24^ Community engagement and prioritisation are not always present, particularly in regions where regular power-outages are not common.^2^ This study aimed to identify the worldwide barriers and facilitators to implementing on-site hospital solar power, prioritise these determinants and develop a tool containing high value strategies to aid implementation of solar power systems.

## Methods

### Ethics approval

Not applicable. This study did not involve patient data and therefore formal ethical approval was not required.

### Overview of study design

**Figure 1:**
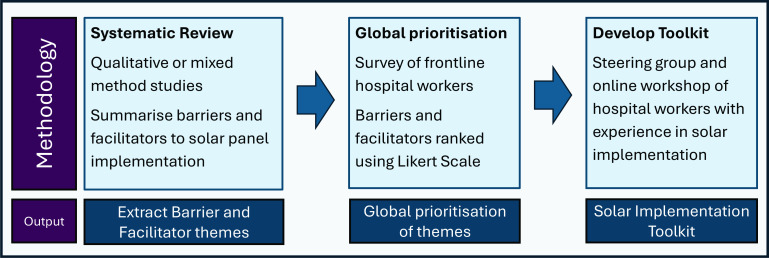
Three-phases of the research methodology.

A three-phase methodology was designed (figure 1). Phase 1 summarised existing barriers and facilitators from a systematic review of academic literature on hospital solar power implementation and mapped these to the Consolidated Framework for Implementation Research (CFIR). The CFIR provided a flexible, context-sensitive structure for organising and interpreting the findings. Phase 2 prioritised the identified barriers and facilitators in a global survey of frontline hospital workers who ranked each determinant using a Likert scale. This approach grounded the results in practical, real-world perspectives and helped identify globally relevant priorities. Phase 3 developed implementation strategies to overcome the barriers and enhance facilitators. These strategies were consolidated into a dedicated solar implementation tool (SOLAR-IT), designed to support and streamline future solar power adoption in hospital settings. Detailed methods for each phase are shown below. As this research involved international partnerships between high-income countries (HICs) and LMICs, a reflexivity statement has been completed (online supplemental S1).

### Phase 1: systematic review

A systematic review was conducted to identify existing barriers and facilitators to healthcare solar power implementation. This systematic review was designed following the Preferred Reporting Items for Systematic Reviews and Meta-Analysis (PRISMA) guidelines (online supplemental S2). A protocol was not published. This systematic review was registered with PROSPERO (CRD42024599092).

#### Eligibility criteria

Inclusion criteria were published qualitative and mixed method studies, letters, literature reviews and reports from national/international organisations set in LMICs, focused on on-site solar power implementation in hospitals, healthcare facilities or primary care facilities (online supplemental table S1). Exclusion criteria included abstracts, protocols or registered studies set in HICs with a focus on non-healthcare settings, population health or medical devices and studies that did not have a qualitative component. Studies set in HICs, including rural or remote regions, were excluded as determinants are likely shaped by different systems than LMICs, such as baseline electricity supply, financial opportunities and technological availability. These may not be relevant in LMIC settings and could bias the list of determinants and limit interpretability of the final list of barriers and facilitators for decision-makers. Studies older than 15 years were excluded, due to the significant advances in solar power technology over this time.^25^

#### Information sources

A systematic search was performed from 25 October 2024 to 05 November 2024 of the MEDLINE, WHO Institutional Repository for Information Sharing (IRIS), Cochrane, Embase, Web of Science, JSTOR, Scopus, EBSCO and World Bank Open Knowledge Repository databases. All languages were included. A grey literature search using search engines (eg, Google) and targeted website searching was not performed. We conducted backward citation tracking to identify additional relevant studies for inclusion.

#### Search strategy and selection process

Search terms were mapped to the domains ‘population’, ‘intervention’ and ‘setting’, combined using the Boolean operator (AND) (online supplemental table S2). Within each domain, search terms were combined using the Boolean operator (OR). Medical Subject Headings (MeSH) were identified. A sample search strategy for PubMed is available in online supplemental S3-S9. To minimise bias, articles were independently screened by two individuals (CL and RT) and assessed using EndNote. Discrepancies were discussed with a third individual (DN). During article selection, any abstracts identified not in English were translated using Google Translate. Duplicate studies were removed using EndNote.

#### Data extraction

Data was extracted by two individuals independently (CL and RT), and disagreements resolved through discussion with the senior author (DN). All articles included were in English, so additional translation was not required. Data for each article was extracted into Microsoft Excel and included study name, author, date, journal, geographical location researched, component of health facility assessed, health facility type, number of hospitals addressed and whether these were rural or urban locations.

#### Data synthesis

Included studies had already used a component of qualitative methodology to identify barrier or facilitator factors. Relevant factors were extracted from the papers and categorised into ‘barrier’ or ‘facilitator’. Within each category, factors were iteratively summarised independently by CL and RT, and discrepancies resolved by DN. Themes were then mapped to domains from the CFIR.^26^ The CFIR is a determinants framework that aids implementation by providing a standardised and methodical structure for organising data. It was selected for this study due to its contextual sensitivity and ability to facilitate cross-disciplinary action. The CFIR consists of five domains (innovation, outer setting, inner setting, individual and implementation process), within which are constructs. A steering group of researchers from the National Institute for Health and Care Research (NIHR Research Unit on Global Surgery defined each domain; ‘innovation domain’ was defined as ‘decentralised solar power and battery systems’, ‘external domain’ as ‘international and national agencies’, ‘internal domain’ as the ‘local hospital community’ and ‘individual domain’ defined as the ‘hospital workers’. Barriers and facilitators were subsequently assigned to each CFIR domain. Inductive domains were additionally assigned by the steering committee, to increase usability of the determinants for those unfamiliar with the CFIR.

#### Quality appraisal

Scoping review prior to this study demonstrated heterogeneity in methodology with very few papers reporting robust qualitative methodology. Formal quality appraisal at this stage risked excluding potentially important determinants in a field with limited evidence. Therefore, we did not perform quality appraisal and instead used the systematic review to synthesise an exhaustive list of barriers and facilitators. These determinants were then prioritised in the real world using a survey of front-line global hospital workers. This allowed grounded and contextual appraisal of each barrier and facilitator to solar implementation.

### Phase 2: global prioritisation survey

#### Survey

To understand how global hospital workers prioritise the barriers and facilitators to solar implementation, an online survey was created using the Research Electronic Data Capture web application (Vanderbilt University, Nashville, Tennessee, USA). This allowed a contextual framing of the implementation determinants by frontline workers across multiple geographical and political climates. All barrier and facilitator factors identified from the systematic review were included in the survey. Participants were asked to rank the barrier and facilitator factors on a 5-point Likert scale from ‘Very Unimportant’ to ‘Very Important’. An ‘other’ option was available for participants, which included free-text response if the relevant barrier was not listed.

The survey was distributed to a network of healthcare clinicians, technicians, engineers and managers via the NIHR Global Health Research Unit on Global Surgery network. This included participants who had successfully participated in a previous survey on healthcare power-outages, so had familiarity with healthcare facility electricity infrastructure. Participants were encouraged to discuss with relevant hospital managers or technicians if they were not familiar with infrastructure projects in their hospital. Participants from all income groups were invited to complete the survey. We included HIC respondents as a secondary comparator group to explore how prioritisation of determinants differed between HICs and LMICs. This helps interpret which barriers and facilitators are universal and which are context specific to LMICs, enabling policymakers to support more targeted and appropriate implementation strategies in LMICs.

#### Statistical analysis

Primary analysis included only respondents from LMICs, as the target population. Secondary analysis included respondents from HICs. Statistical analysis was performed in R. Likert responses were treated as categorical values.^27^ Results were described as the proportion of respondents who had positively ranked an implementation factor (ranked as either ‘important’ or ‘very important’). To calculate differences in responses between LMIC and HIC hospital workers, proportion of LMIC positively ranked factors was subtracted from proportion of HIC positively ranked factors. Income group was classified using World Bank definitions.

### Phase 3: development of the solar implementation tool

The final phase was to create a tool that could be used to aid rapid implementation of decentralised healthcare solar power. This solar implementation tool (SOLAR-IT) was co-developed by a steering group formed of international hospital workers and engineers with experience in healthcare solar power implementation. The steering group had representation from low-income, middle-income and HICs and clinical and engineering backgrounds. This team was selected as it already had experience in implementing three hospital solar implementation projects (Nigeria, India and Uganda, online supplemental S10). This ensured that members of the group had diverse and grounded perspectives to bring to the development of the SOLAR-IT.

A facilitated discussion was conducted with the steering group to create the SOLAR-IT. Strategies were iteratively co-developed. First, the results of the systematic review and survey were reviewed and summarised. Barriers were assigned to relevant facilitators through group discussion, and enabling strategies were co-formulated using an iterative process, discussing each implementation factor across the group. The SOLAR-IT was represented to the group and refined until agreement was reached among all group members.

Real-world experiences from the three solar panel implementation projects were documented by the steering group (online supplemental S10). The SOLAR-IT was discussed within the setting of these case-studies to ensure that the strategies identified could be contextually relevant and exhaustive.

### Patient and public involvement

Patients and the public were not involved in the design, conduct, reporting or dissemination of this research.

## Results

### Phase 1: systematic review

**Figure 2:**
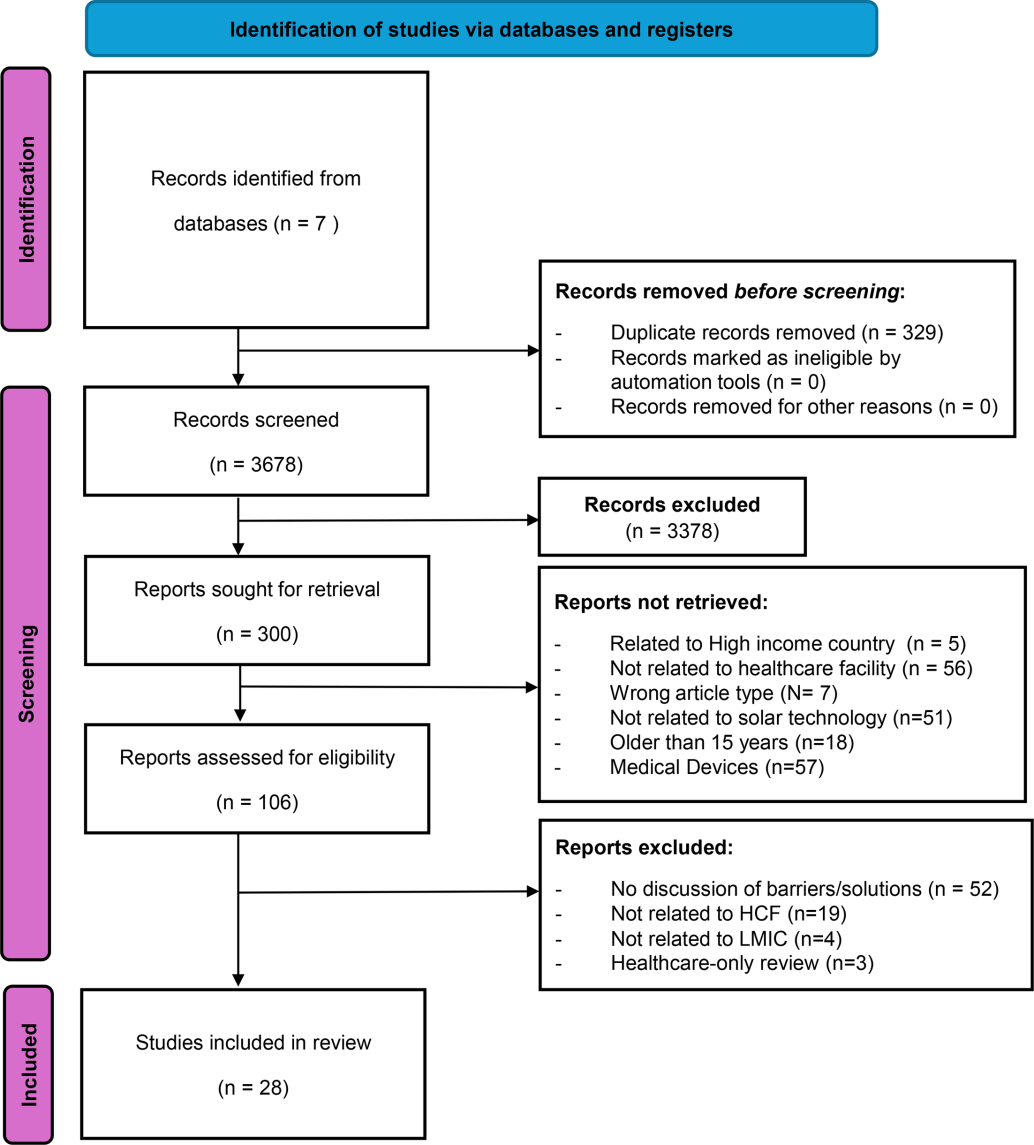
PRISMA flow chart of included and external studies for the systematic review. HCF, Health Care Facilities; LMIC, low- and middle-income country; PRISMA, Preferred Reporting Items for Systematic Reviews and Meta-Analysis.

Search results returned 3678 individual records, of which 106 were included for full-text review and 28 fulfilled inclusion criteria (figure 2). Of these, nine were qualitative studies analysing data from interviews, narrative descriptions of implementation or expert opinion about solar panel implementation (table 1). There were 19 mixed-methods studies, which included qualitative interviews, narrative reviews of healthcare and non-healthcare studies, expert opinion, policy analysis and patient or staff surveys. Of the papers selected, 15 papers were based in single countries, 5 included multiple sub-Saharan Africa countries, 8 had global perspectives. Half of the papers were published in non-healthcare journals (namely engineering, energy and geography journals).

**Table 1:** Papers included in the systematic review

Author	Date	Location	Study design	Study aims
Jacobus *et al*	2011	Sierra Leone	Mixed methods: modelling and observations	Evaluate techno-economic hybrid solar power for primary health facility
WHO	2014	Global	Mixed methods: narrative review, policy analysis	Report on global healthcare electrification implementation
Franco *et al*	2017	Global	Mixed methods: modelling and narrative review	Estimate energy requirements, describe determinants of electrification solutions
Duke *et al*	2017	Papua New Guinea	Mixed methods: observations, modelling	Describe large-scale implementation of solar-power oxygen to healthcare facilities
Ramji *et al*	2017	India	Qualitative: in-depth interviews	Assess how electricity impacts healthcare delivery, investigate determinants of off-grid healthcare solar power
Swanson *et al*	2017	DRC, Pakistan, Kenya, Zambia	Qualitative: narrative description	Describe implementation of an ultrasound clinic
Babatunde *et al*	2018	Nigeria	Mixed methods: modelling, survey and narrative review	Evaluate techno-economic stand-alone solar power for primary health facility
Ignjatovic *et al*	2018	Equatorial Guinea	Mixed methods: narrative review	Provide guidelines for healthcare facility design in LMICs
Dholakia *et al*	2018	Global	Qualitative: narrative review, observations, expert opinion	Describe global challenges of implementing solar power in healthcare
Reuland *et al*	2019	Malawi	Mixed methods: in-depth interviews, staff survey	Assess impacts of solar power implementation on healthcare and environment
Opoku *et al*	2020	Ghana	Mixed methods: qualitative survey	Describe access to electricity affected healthcare service delivery and rural development
Javadi *et al*	2020	Ghana, Uganda	Qualitative: in-depth interviews	Assess impact of healthcare solar power, identify implementation factors
Agbo *et al*	2021	Nigeria	Mixed methods: modelling and narrative review	Review innovative solar technology in Nigeria, estimate expansion across the country
Moner-Girona *et al*	2021	SSA	Mixed methods: modelling and observations	Assess healthcare electrification, estimate techno-economic impact of solar power
Pakravan *et al*	2021	Uganda	Mixed methods: observations, survey, policy analysis	Develop toolkit to allocate healthcare electricity investment
Pulsan *et al*	2021	Papua New Guinea	Qualitative: in-depth interviews	Explore views of health workers on the impact of electrification on health facilities
Ngoh *et al*	2022	Cameroon	Mixed methods: modelling and narrative discussion	Evaluate techno-economic stand-alone solar power for primary health facility
Soto *et al*	2022	Global	Mixed methods: narrative review	Describe implementation factors of healthcare solar power
Olatomiwa *et al*	2022	SSA	Mixed methods: narrative review, policy analysis	Describes solar power solutions for rural healthcare facilities and discusses benefits
Paim *et al*	2022	Nigeria	Qualitative: in-depth interviews	Investigate business models for implementing sustainable healthcare electrification
Izuka *et al*	2023	Global	Mixed methods: narrative review	Describes solar power solutions for rural healthcare facilities and discusses benefits
WHO	2023	Global	Mixed methods: narrative review, policy analysis, expert opinion	Report on global healthcare electrification implementation
WHO	2023	SSA	Qualitative: in-depth interviews, case studies, expert opinion	Track global healthcare electrification, recommendations on transition to renewable energy
Felice *et al*	2023	Uganda	Qualitative: in-depth interviews	Investigate feasibility of replacing emergency diesel generators with solar power
Dehshiri *et al*	2024	Iran	Mixed methods: narrative review, observations and modelling	Model techno-economic impact of healthcare facility solar power in five different climate regions
Sharma *et al*	2024	Global	Mixed methods: narrative review, policy analysis, expert opinion	Report on global healthcare electrification
WHO	2024	Global	Mixed methods: narrative review, policy analysis, expert opinion	Report on global healthcare electrification implementation
Glas *et al*	2024	Malawi	Qualitative: narrative description	Describe solar panel implementation

DRC, Democratic Republic of the Congo; LMICs, low- and middle-income countries; SSA, sub-Saharan Africa.

In total, 19 barriers and 29 facilitator factors on solar panel implementation were summarised (online supplemental table S3). The most commonly described barriers were the high upfront cost of solar panels (n=12 papers), and the requirement for skilled workers to complete installation (n=11 papers). Most discussed facilitators included community and staff training, shifting non-critical loads to non-peak hours, multinational partnerships, government advocacy for solar panels and remote energy assessments using modelling software (n=7 papers for each) (online supplemental table S4).

**Figure 3:**
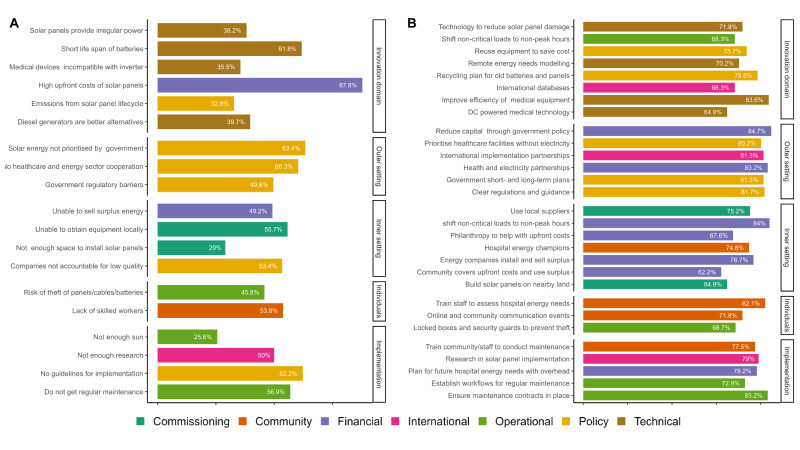
Proportion of positively rated responses (‘important’ or ‘very important’) for (A) barriers and (B) facilitators, structured using the Consolidated Framework for Implementation Research and coloured by inductive domain. DC, Direct Current

The barriers and facilitators were mapped to the CFIR (figure 3). Within the innovation domain, barriers were mainly technical (eg, short battery lifespan, reliance on diesel alternatives), addressed through technical facilitators, such as more efficient equipment and policy facilitators such as equipment reuse and recycling. In the outer setting, barriers were largely policy-related, including poor inter-sector cooperation, lack of prioritisation and government regulation. These were matched through policy facilitators, such as government planning and regulation, as well as financial mechanisms to reduce capital expenditure. Inner setting barriers included commissioning barriers (eg, difficulty obtaining local equipment), financial constraints (eg, inability to sell surplus energy) and regulatory challenges. Most inner setting facilitators included local and community partnerships to reduce capital expenditure. At the individual level, the risk of theft and lack of skilled local workers were barriers to solar power implementation. These can be addressed by using locked boxes to prevent theft and training local communities in solar power implementation and maintenance. Finally, within the implementation domain, barriers included lack of research and lack of maintenance, while implementation facilitators included community action, such as training staff to conduct maintenance and operational facilitators such as maintenance workflows and contracts.

### Phase 2: global prioritisation survey

The survey was completed by 260 individual LMIC hospital workers and 221 HIC hospital workers. There were 22 duplicate responses removed. There were 206 hospitals represented from 47 different LMIC countries (online supplemental figure S1). Of the LMIC respondents, most were doctors (89.2%, n=232), of which 140 (60.3%) sought help from managers, clinical leads, electricians or other administrators in answering the survey. There were 107 (41.1%) respondents from upper middle-income countries, 132 (50.8%) from lower middle-income countries and 21 (8.1%) were from low-income countries (online supplemental table S5). Most respondents (77.7%, n=202) were from tertiary level hospitals. Just over half (134, 51.5%) of hospitals were national government hospitals, and 79 (30%) were state/local government hospitals. Breakdown of HIC respondents as a secondary comparator group are shown in online supplemental table S5.

Of the LMIC respondents, 106 (40.8%) reported that solar power was already installed in their hospitals, 99 (96.1%) of which were working. In 18 (11.7%) LMIC hospitals, a previous unsuccessful attempt had been made to instal solar panels, 11 of which were abandoned at the initial planning stage, 3 failed during solar panel installation and 3 failed after implementation. There were 15 free-text responses (5.7% of LMIC respondents) describing reasons for failure. These have been summarised and include changes in financial costs and funding (n=8, 53%), corruption risks (n=1, 6.6%), inadequate support from government (n=2, 13.3%), war (n=1, 6.6%) and lack of space (n=1, 6.6%). Future solar installation was planned in 80 (38.8%) hospitals.

The percentage of positively ranked barriers (ranked ‘important’ or ‘very important’) varied from 26% (‘not enough sun’) to 88% (‘high upfront costs of solar panels’) (figure 3A). Other highly prioritised barriers included ‘solar energy not prioritised by government’ (63%), ‘no guidelines for implementation’ (62%), ‘short life span of batteries’ (62%) and ‘no healthcare and energy sector cooperation’ (60%). Of the 21 respondents who filled out ‘other’, there were no new barriers identified.

There was more homogeneity between positively ranked facilitators, from 62% (‘community covers upfront costs and uses surplus energy’) to 85% (‘reduce capital through government policy’) (figure 3B). Further important facilitators were ‘Fund installation within larger infrastructure projects’ (84%), ‘Improve efficiency of medical equipment’ (84%), ‘Health and energy partnerships’ (83%) and ‘Ensure maintenance contracts in place’ (83%). Of the 21 (8%) other responses, no new barriers were identified.

Secondary analysis demonstrated five barriers prioritised by both HICs and LMICs, with less than 2% difference between income groups. These were incompatible medical devices, emissions from the solar panel life-cycle, alternative energy sources, low government prioritisation and limited healthcare and energy sector cooperation (online supplemental figure S1). Respondents in LMIC were more likely to find lack of local equipment and skilled workers as important barriers. HIC hospital workers found limited space for solar panels and inadequate solar irradiance as key barriers. Nearly all facilitators were considered more feasible in LMICs (online supplemental figure S2).

### Phase 3: development of the solar implementation tool

**Figure 4:**
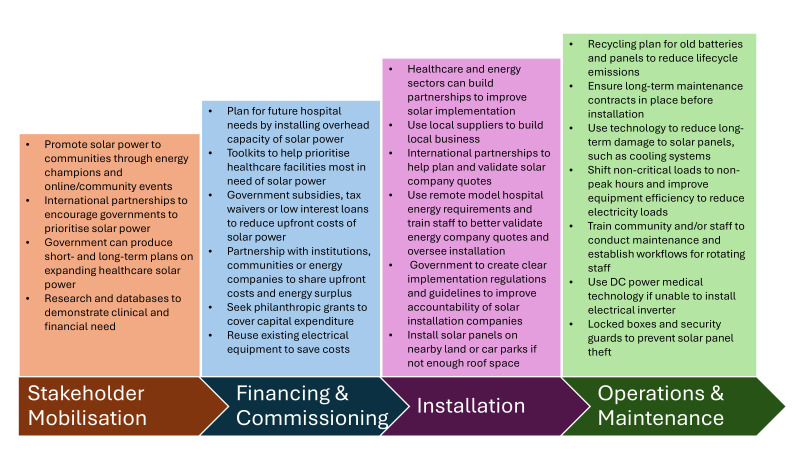
The SOLAR-IT, a tool containing strategies for implementing decentralised solar power in low- and middle-income country healthcare facilities. DC, Direct Current.

The steering group linked the barriers and facilitators (online supplemental figure S3) and identified 23 implementation strategies from the barriers and facilitators. These were grouped into four higher-level themes (stakeholder mobilisation, commissioning, installation and operations and maintenance) to reflect the sequential steps of implementing on-site hospital solar power. These strategies are presented as the SOLAR-IT (figure 4).

## Discussion

This study summarises common barriers and facilitators to on-site healthcare facility solar power through a systematic literature review and prioritises these across a global network of clinicians and hospital workers. We identified multiple barriers and facilitators, with cost and government action prioritised as the most important factors. These determinants are consistently linked across multiple domains, requiring coordinated solutions. Strategies to bridge these implementation factors have been synthesised into the solar implementation tool SOLAR-IT, which can help healthcare teams across the world rapidly deploy decentralised solar systems.

Power-outages remain common and devastating events in many LMIC hospitals.^28–30^ Solar power is a less polluting, quick to instal and often more reliable solution than expansions to the electrical grid. Expanding on-site solar system implementation, either as an off-grid or hybrid solution, is an urgent priority to strengthen energy security and decarbonise hospitals. The Lancet Countdown on climate change advocates healthcare facilities to transition to renewable electricity sources as essential in mitigating climate change.^9^ Despite this urgent necessity, there is limited research on solar power utilisation in healthcare. Additionally, half of the papers found were in non-healthcare journals, limiting accessibility on healthcare search platforms. This siloed approach to healthcare electricity research has likely limited data collection and research to date.^2 31 32^

Intersectoral cooperation was an underlying theme across results. Implementing on-site solar hospital solar power is complex and requires diverse and locally adapted solutions. Multidisciplinary team action supports almost all strategies of the SOLAR-IT. Case studies from sub-Saharan Africa demonstrate how international partnerships can overcome financial and installation barriers.^33^ Collecting data and building networks with energy companies is essential to prioritise facilities with the greatest electricity inequality, ensure supply chains are sustainable and provide long-term sustainability and maintenance.^34 35^ Public–private partnerships may help with the development of appropriate technology, such as Direct Current (DC) powered equipment or more environmentally resilient solar panels. Waste from batteries and solar panels was highly prioritised by the literature; public–private partnerships can ensure that robust recycling and waste management plans are established.^36^

Additionally, building local partnerships with companies can develop local skillsets and trust.^24 37^ Unreliable local companies delayed implementation in both our case studies and for a hospital in Malawi; stricter government regulation can ensure higher quality of local services.^33^ Local engagement of public, hospital staff, local business and government is essential to successful solar power implementation. Lack of community engagement was identified as a barrier in several studies.^2 6 24 34 38 39^ The perceived importance of implementing secure, decentralised electricity is likely higher in hospitals experiencing frequent power outages, while for regions with stable and lower-cost electricity, solar adoption may be a lower priority for communities and staff. Successful projects in healthcare and other sectors engage communities early and build support to navigate barriers to solar implementation.^24 33 40 41^ Building local expertise and training healthcare staff to assist with implementation and maintenance can reduce costs and improve sustainability.^2 33^ Healthcare facilities can act as an epicentre of infrastructure development, sharing upfront costs of solar power, then distributing surplus to the community, as has been achieved in other sectors.^40^

High capital expenditure of solar panel installation was the most prioritised barrier to implementation in LMICs. International aid to Africa can help overcome high costs, and has been shown to foster growth in renewable energies over fossil fuels.^42^ Sharing upfront costs with larger institutions, such as universities and government buildings is a possible solution for urban healthcare facilities.^39^ However, this is less likely to be feasible in rural areas, where surrounding infrastructure is limited but power is mostly needed. Our survey showed hospital workers consider government action to be the highest priority in reducing costs and facilitating implementation. Government can have an essential role in reducing up-front costs in many countries.^6 32^ Effective policies can include subsidies, tax waivers on solar panel imports, market-oriented financing and declining alternative compliance payment rates.^1 32 39^

The cost of mains electricity can place financial strains on healthcare systems; for example, one hospital in Nigeria has been without electricity for 100 days due to outstanding debt to the government electricity company.^43 44^ Furthermore, diesel generators require continuous fuel procurement, making them susceptible to price volatility.^35^ Solar power has low operating costs and can either replace or supplement mains and diesel generator electricity, lowering long-term expenditure. While the upfront costs of solar power are high, they are rapidly decreasing and long-term cost savings can offset the capital investment.^12 13 22 45 46^ This is supported by techno-economic studies, demonstrating how hybrid or off-grid solar power can be cost-effective for hospitals.^13–21^ Modelling software, such as HOMER Software (UL Solutions, Boulder, Colorado, USA), can help hospitals estimate long-term finances and provide business cases for investment in solar power.^47^

Some barriers identified to healthcare solar implementation are shared across LMIC and HIC settings and may be overcome through global collaboration and action. For example, research to improve solar system lifecycles and equipment compatibility could help implementation across all countries. However, most barriers and facilitators identified in this study are specific to LMICs and require locally adaptable strategies. The SOLAR-IT is a structured implementation tool that can help policymakers navigate these barriers and facilitators. It includes both context-specific strategies (eg, use of locked boxes to prevent theft) and universal strategies (eg, remote modelling software for system planning), making it flexible across different settings. In practice, the SOLAR-IT can be used alongside local resources needed to implement solar power, including sustainable financing for capital expenditure and maintenance, a multidisciplinary implementation team, governance arrangements for commissioning and technical expertise for installation.

There are limitations to this study. The barriers and facilitators identified from the systematic review can be context specific. To enhance the validity of these determinants in other settings, we used the CFIR, which helps structure determinants in context and the survey of frontline hospital workers to obtain grounded and real-world priorities. We did not undertake a formal quality appraisal, as our aim was to generate an exhaustive list of barriers and facilitators and there were few high-quality implementation studies. Quality appraisal at this stage risked excluding important determinants. We subsequently prioritised each determinant through a global survey of frontline hospital workers, providing real-world assessment of the perceived importance of each barrier and facilitator. The survey was disseminated through the Global Surgery Unit network. This network has major strengths, with a reach of more than 20 000 recipients. However, it may under-represent rural primary care facilities, which may have different infrastructure and organisational priorities, limiting the transferability of results across all LMIC settings. Furthermore, the network is mostly comprised of clinicians, resulting in few non-clinician responses to the survey. Clinicians may have incomplete information on technical issues or operational planning. We sought to mitigate this by encouraging clinicians to discuss determinants with their local technical personnel. Half of clinicians sought advice from another hospital member; however, clinicians in remote centres with limited resources may have wider background understanding of infrastructure issues than clinicians in complex urban hospitals. The survey demonstrated homogeneity of responses, particularly when ranking facilitators. This may indicate equal prioritisation of all themes, or that respondents lack experience to differentiate between themes. Finally, our steering and writing committee had limited representation from technical disciplines such as engineering and energy policy. However, this was partly mitigated by including researchers who are leading experts in hospital system design, implementation research and hospital managers with experience in infrastructure projects. Furthermore, in India, Mexico and Nigeria, where power outages are common and repeatedly interrupt care, clinicians are actively engaged in supporting electricity development and capacity.

Strengthening energy security can help build LMIC health system capacity. However, further work is needed to research the health, carbon and financial impacts of decentralised hospital electricity. The SOLAR-IT would additionally be strengthened by a prospective validation and iterative development through real-world applications. Expanding implementation research on hospital solar power can help strengthen policy advocacy and help mobilise investment.

## Conclusion

This study has identified solar power implementation factors from the literature, prioritised these through consulting front-line hospital workers, and developed the SOLAR-IT. We provide the first global overview of solar panel implementation in healthcare and contribute to a new body of work on securing and decarbonising healthcare electrification. The SOLAR-IT could be an essential tool to aid rapid expansion of decentralised healthcare electricity. Further research is needed to prospectively study implementation of on-site healthcare facility solar power and build understanding of the tool in practice.

## Supplementary material

10.1136/bmjgh-2026-023926 online supplemental file 1

10.1136/bmjgh-2026-023926 online supplemental file 2

10.1136/bmjgh-2026-023926 online supplemental file 3

10.1136/bmjgh-2026-023926 online supplemental file 4

10.1136/bmjgh-2026-023926 online supplemental file 5

## Data Availability

Data are available upon reasonable request.
