# Supplementary material for: Barriers, facilitators and strategies for implementing on-site hospital solar power in low- and middle-income countries: a systematic review, global prioritisation survey and development of an implementation tool
Source: BMJ Glob Health. 2026 Jun 18;11(6):e023926. doi: 10.1136/bmjgh-2026-023926 (PMC13288949; doi:10.1136/bmjgh-2026-023926)
Supplement: online supplemental file 1 [file bmjgh-11-6-s001.docx]

### Supplementary S1: BMJ Global Health Author Reflexivity Statement

Adapted from Morton, B., Vercueil, A., Masekela, R., Heinz, E., Reimer, L., Saleh, S., Kalinga, C., Seekles, M., Biccard, B., Chakaya, J., Abimbola, S., Obasi, A. and Oriyo, N. (2022), Consensus statement on measures to promote equitable authorship in the publication of research from international partnerships. Anaesthesia, 77: 264-276. <https://doi.org/10.1111/anae.15597>

| **Study conceptualisation** | |
| --- | --- |
| 1. How does this study address local research and policy priorities? | This paper focuses on energy security in LMICs, a topic highlighted by the WHO, world bank and UN as critical to enabling healthcare. This also fits with sustainable development goals 3 (health and wellbeing), 7 (affordable and clean energy) and 13 (climate action). Research priorities were defined through consultation with LMIC partners prior to protocol development. To ensure this research addresses local priorities, we prioritised results from the systematic review through an international engagement exercise, involving hospital workers from 47 LMIC countries. The final implementation tool is designed to be flexible and locally adaptable. |
| 1. How were local researchers involved in study design? | The study was co-led by researchers in Nigeria, India and Mexico. Study design, analysis of results and review of paper was conducted jointly with these authors. The advisory and writing group (with 9 LMIC researchers) helped conceptualise and design this study and were involved throughout each step. |
| **Research management** | |
| 1. How has funding been used to support the local research team(s)? | Members of the advisory and writing group are funded through NIHR156087: NIHR Group on Environmentally Sustainable Hospitals in Low and Middle Income Countries. This group operates through a hub and spoke network, with funded “hub” in Nigeria, Mexico, Ghana, India, South Africa, Rwanda and Benin. Each hub has a network of “spoke” hospitals within the country to mobilise researchers. The group is equitable and research is co-developed and produced. Respondents of the survey were not funded. |
| **Data acquisition and analysis** | |
| 1. How are research staff who conducted data collection acknowledged? | All research staff who conducted data collection are acknowledged as NIHR Group on Environmentally Sustainable Hospitals in Low- and Middle-Income Countries in the authorship, with names and institutions in the supplement. |
| 1. How have members of the research partnership been provided with access to study data? | Data has been summarised and presented to the advisory group, where it was discussed and data analysis planned. |
| 1. How were data used to develop analytical skills within the partnership? | Through group interpretation and analysis of data there was knowledge sharing between researchers of different income groups. |
| **Data interpretation** | |
| 1. How have research partners collaborated in interpreting study data? | Summarised data from the systematic review and the global prioritisation exercise was presented to the advisory group. The group then made decisions on how to interpret the data. The group used the data to create the SOLAR-IT, iteratively reviewing responses from the global prioritisation exercise and developing strategies from the barriers and facilitators.  Data interpretation was strengthened by the mix of disciplines and backgrounds of participants |
| **Drafting and revising for intellectual content** | |
| 1. How were research partners supported to develop writing skills? | Initial draft was written by CL. The advisory and writing group reviewed and iteratively improved the initial draft |
| 1. How will research products be shared to address local needs? | The SOLAR-IT has already been used in implementing solar power in a hospital in Nigeria. The advisory group is working to build policy capacity in several LMICs to disseminate the tool and facilitate implementation of solar power. |
| **Authorship** | |
| 1. How is the leadership, contribution and ownership of this work by LMIC researchers recognised within the authorship? | LMIC researchers AA, DG, AS, DS, PH, JA, AR are included in the topline authorship. All contributors to the survey or advisory group are recognised through the NIHR Group on Environmentally Sustainable Hospitals in Low- and Middle-Income Countries |
| 1. How have early career researchers across the partnership been included within the authorship team? | CL, RT are early career researchers. CL co-led design, analysis and write up, while RT was a second reviewer for the systematic review. |
| 1. How has gender balance been addressed within the authorship? | Four members of the advisory group were women |
| **Training** | |
| 1. How has the project contributed to training of LMIC researchers? | The study was co-led by LMIC researchers, and the advisory group facilitated knowledge sharing between researchers of different disciplines and income groups |
| **Infrastructure** | |
| 1. How has the project contributed to improvements in local infrastructure? | This project aims to improve local infrastructure, through creation of an implementation tool to enable on-site hospital solar power. This has already been used to implement solar power in a Nigerian hospital, and we are planning to expand this to implement solar power in more LMIC hospitals. |
| **Governance** | |
| 1. What safeguarding procedures were used to protect local study participants and researchers? | This study did not collect patient data. All respondents of the survey were informed of intention to publish results and provided implied consent in completing the survey. |
