# Supplementary material for: Barriers, facilitators and strategies for implementing on-site hospital solar power in low- and middle-income countries: a systematic review, global prioritisation survey and development of an implementation tool
Source: BMJ Glob Health. 2026 Jun 18;11(6):e023926. doi: 10.1136/bmjgh-2026-023926 (PMC13288949; doi:10.1136/bmjgh-2026-023926)
Supplement: online supplemental file 3 [file bmjgh-11-6-s003.docx]

**Supplementary S3: MEDLINE Search Strategy**

Date: 10^th^ October 2024

(("Hospitals"[Mesh]) OR ("Health Facilities"[Mesh]) OR (clinic) OR (Hospitals))

AND

("Solar Energy"[Mesh]) OR (“Solar Power”) OR (“Solar Panel”) OR (“Photovoltaic”) OR (“Solar Cell*”)

AND

(("Developing Countries"[Mesh]) OR (("developing countr*") OR ("developing nation*") OR ("developing world") OR ("less developed countr*") OR ("less developed nation*") OR ("less developed world") OR ("lesser developed countr*") OR ("lesser developed nation*") OR ("under developed countr*") OR ("under developed nation*") OR ("under developed world") OR ("underdeveloped countr*") OR ("underdeveloped nation*") OR ("underdeveloped world") OR ("middle income countr*") OR ("middle income nation*") OR ("middle income population*") OR ("low income countr*") OR ("low income nation*") OR ("low income population*") OR ("lower income countr*") OR ("lower income nation*") OR ("lower income population*") OR ("underserved countr*") OR ("underserved population*") OR ("under served population*") OR ("under served nation*") OR ("under served population*") OR ("deprived countr*") OR ("deprived population*") OR ("high burden countr*") OR ("high burden nation*") OR ("countdown countr*") OR ("poor countr*") OR ("poor nation*") OR ("poor population*") OR ("poor world") OR ("poorer countr*") OR ("poorer nation*") OR ("poorer population*") OR ("developing econom*") OR ("less developed econom*") OR ("underdeveloped econom*") OR ("under developed econom*") OR ("middle income econom*") OR ("low income econom*") OR ("lower income econom*") OR ("low gdp") OR ("low gnp") OR ("low gross domestic") OR ("low gross national") OR ("lower gdp") OR ("lower gross domestic") OR ("lmic") OR ("lmics") OR ("third world") OR ("lami countr*") OR ("transitional countr*") OR ("emerging econom*") OR ("emerging nation*")) OR ("afghan*" OR "africa*" OR "albania*" OR "algeria*" OR "angola*" OR "argentin*" OR "armenia*" OR "azerbaijan*" OR "bangladesh" OR "bengal*" OR "bangal*" OR "belarus*" OR "belorus" OR "byelarus" OR "byelorus" OR "belize" OR "benin" OR "dahomey" OR "bhutan" OR "bolivia*" OR "bosnia*" OR "herzegovia" OR "botswan*" OR "batswan*" OR "bechuanaland*" OR "brazil*" OR "brasil*" OR "bulgaria" OR "burkina*" OR "burkinese" OR "upper volta*" OR "burundi*" OR "urundi*" OR "cabo verde" OR "cape verde" OR "cambodia*" OR "kampuchea*" OR "khmer" OR "cameroon" OR "cameroun" OR "ubangi shari*" OR "chad*" OR "colombia*" OR "comoro" OR "comore" OR "comorian" OR "mayotte*" OR "congo*" OR "zaire*" OR "costa rica*" OR "cote d'ivoir*" OR "cote d' ivoir*" OR "cote divoir*" OR "cote d ivoir*" OR "ivory coast" OR "ivorian*" OR "cuba" OR "cuban" OR "cubans" OR "cuba's" OR "djibouti" OR "french somaliland" OR "dominica*" OR "ecuador*" OR "egypt*" OR "united arab republic*" OR "el salvador" OR "salvadoran" OR "guinea*" OR "guinea" OR "eritrea*" OR "eswatini*" OR "swaziland" OR "swazi*" OR "swati*" OR "ethiopia*" OR "fiji" OR "gabon*" OR "gabonese*" OR "gabonaisse" OR "gambia*" OR ("georgia" OR "georgian" OR "georgians") NOT ("atlanta" OR "california" OR "florida") OR "ghana" OR "grenada" OR "grenadian" OR "guatemala" OR "guyana" OR "guiana*" OR "guyanese" OR "haiti*" OR "hispaniola*" OR "hondura*" OR "india*" OR "indonesia*" OR "iran*" OR "iraq*" OR "jamaica*" OR "jordan*" OR "kazakh*" OR "kenya*" OR "karabati*" OR "korea*" OR "kosovo" OR "kosova*" OR "kyrgyz*" OR "kirgiz*" OR "kirghiz*" OR "laos" OR "lao" OR "laotian*" OR "lebanon" OR "lebanese" OR "lesotho*" OR "lesothan" OR "lesothonian" OR "basutoland*" OR "mosotho" OR "basotho" OR "liberia*" OR "libya*" OR "jamahiriya" OR "macedonia*" OR "madagascar" OR "malagasy" OR "malawi*" OR "nyasaland" OR "malaysia*" OR "malay*" OR "maldives" OR "maldivian*" OR "indian ocean" OR "mali*" OR "micronesia*" OR "marshallese" OR "kiribati" OR "marshall island" OR "tuvalu" OR "mauritania*" OR "maurit*" OR "mexico*" OR "mexican*" OR "moldova*" OR "moldovia*" OR "mongol*" OR "morocco*" OR "moroccan*" OR "ifni" OR "mozambique" OR "mozambican*" OR "myanmar*" OR "burma" OR "burmese" OR "namibia*" OR "nepal*" OR "netherlands antill*" OR "nicaragua*" OR "niger*" OR "omani" OR "omanis" OR "oman's" OR "pakistan*" OR "palestin*" OR "gaza*" OR "west bank*" OR "panama" OR "paraguay" OR "peru*" OR "philippin*" OR "philipin*" OR "phillipin*" OR "filipino*" OR "filipina*" OR "rwanda" OR "rwandese" OR "ruanda" OR "ruandese" OR "samoa*" OR "pacific island*" OR "polynesia*" OR "sao tome and principe" OR "sao tomean*" OR "santomean" OR "senegal*" OR "serbia*" OR "sierra leone" OR "melanesia" OR "solomon island" OR "somali*" OR "sri lanka*" OR "ceylon" OR "saint lucia" OR "st lucia" OR "saint vincent" OR "st vincent" OR "vincentian" OR "grenadine" OR "sudan*" OR "surinam" OR "syria*" OR "tajik*" OR "tadjik*" OR "tadzhik*" OR "tanzania*" OR "tanganyika*" OR "thai*" OR "timor*" OR "togo*" OR "togoles*" OR "togo's" OR "tonga*" OR "tunisia*" OR "turkiy" OR "turkey" OR "turk*" OR "turks" OR "turkish" OR "turkmen*" OR "uganda*" OR "ukrain*" OR "uzbek*" OR "vanuatu*" OR "new hebrides*" OR "venezuela*" OR "vietnam*" OR "viet nam*" OR "yemen*" OR "yugoslav*" OR "zambia*" OR "zimbabwe" OR "rhodesia*" OR "arab*" OR "middle east*" OR "global south*" OR "sahara*" OR "subsahara*" OR "magreb*" OR "maghrib*" OR "west indies*" OR "caribbean*" OR "central america*" OR "latin america*" OR "south america*" OR "central asia*" OR "north asia*" OR "northern asia*" OR "southeastern asia*" OR "south eastern asia*" OR "southeast asia*" OR "south east asia*" OR "west asia*" OR "western asia*" OR "east europe*" OR "eastern europe*") OR ("lower income countries") OR ("low and middle income countries") OR ("LMIC*") OR ("low resource") OR ("global south"))

**Supplementary S4: Embase Search Strategy**

Date: 10^th^ October 2024

1 Solar power.mp.

2 Solar technology.mp.

3 (afghan* or africa* or albania* or algeria* or angola* or argentin* or armenia* or azerbaijan* or bangladesh* or bengal* or bangal* or belarus* or belorus* or byelarus* or byelorus* or belize* or benin* or dahomey or bhutan* or bolivia* or bosnia* or herzegovin* or botswan* or batswan* or bechuanaland* or brazil* or brasil* or bulgaria* or burkina* or burkinese* or upper volta* or burundi* or urundi* or cabo verde* or cape verde* or cambodia* or kampuchea* or khmer* or cameroon* or cameroun* or ubangi shari* or chad* or colombia* or comoro* or comore* or comorian* or mayotte* or congo* or zaire* or costa rica* or "cote d'ivoir*" or "cote d' ivoir*" or cote divoir* or cote d ivoir* or ivory coast* or ivorian* or cuba or cuban or cubans or "cuba's" or djibouti* or french somaliland* or dominica* or ecuador* or egypt* or united arab republic* or el salvador* or salvadoran* or guinea* or equatoguinea* or eritrea* or eswatini* or swaziland* or swazi* or swati* or ethiopia* or fiji* or gabon* or gabonese* or gabonaise* or gambia* or ((georgia or georgian or georgians) not (atlanta or california or florida)) or ghana* or grenada* or grenadian* or guatemala* or guyana* or guiana* or guyanese* or haiti* or hispaniola* or hondura* or india* or indonesia* or iran* or iraq* or jamaica* or jordan* or kazakh* or kenya* or karabati* or korea* or kosovo* or kosova* or kyrgyz* or kirgiz* or kirghiz* or laos or lao or laotian* or lebanon* or lebanese* or lesotho* or lesothan* or lesothonian* or basutoland* or mosotho* or basotho* or liberia* or libya* or jamahiriya* or macedonia* or madagasca* or malagasy* or malawi* or nyasaland* or malaysia* or malay* federation or maldives* or maldivian* or indian ocean or mali or malian* or "mali's" or micronesia* or marshallese* or kiribati* or marshall island* or tuvalu* or mauritania* or mauritan* or mauritius* or mexico* or mexican* or moldova* or moldovia* or mongol* or montenegr* or morocco* or moroccan* or ifni or mozambique* or mozambican* or myanmar* or burma* or burmese or namibia* or nepal* or netherlands antill* or nicaragua* or niger* or omani or omanis or "oman's" or pakistan* or palestin* or gaza* or west bank* or panama* or paraguay* or peru or peruvian* or "peru's" or philippine* or philipine* or phillipine* or phillippine* or filipino* or filipina* or rwanda* or rwandese or ruanda* or ruandese or samoa* or navigator island* or pacific island* or polynesia* or "sao tome and principe*" or sao tomean* or santomean* or senegal* or serbia* or sierra leone* or melanesia* or solomon island* or norfolk island* or somali* or sri lanka* or ceylon* or saint lucia* or st lucia* or saint vincent* or st vincent* or vincentian* or grenadine* or sudan* or surinam* or syria* or tajik* or tadjik* or tadzhik* or tanzania* or tanganyika* or thai* or timor leste* or east timor* or timorese* or togo or togoles* or "togo's" or tonga* or tunisia* or turkiy* or turkey* or turk or turks or turkish or turkmen* or uganda* or ukrain* or uzbek* or vanuatu* or new hebrides* or venezuela* or vietnam* or viet nam* or yemen* or yugoslav* or zambia* or zimbabwe* or rhodesia* or arab* countr* or middle east* or global south or sahara* or subsahara* or magreb* or maghrib* or west indies* or caribbean* or central america* or latin america* or south america* or central asia* or north asia* or northern asia* or southeastern asia* or south eastern asia* or southeast asia* or south east asia* or west asia* or western asia* or east europe* or eastern europe*).mp.

4 (developing countr* or developing nation* or developing population* or developing world or less developed countr* or less developed nation* or less developed world or lesser developed countr* or lesser developed nation* or lesser developed world or under developed countr* or under developed nation* or under developed world or underdeveloped countr* or underdeveloped nation* or underdeveloped world or middle income countr* or middle income nation* or middle income population* or low income countr* or low income nation* or low income population* or lower income countr* or lower income nation* or lower income population* or underserved countr* or underserved nation* or underserved population* or under served population* or under served nation* or under served population* or deprived countr* or deprived population* or high burden countr* or high burden nation* or countdown countr* or countdown nation* or poor countr* or poor nation* or poor population* or poor world or poorer countr* or poorer nation* or poorer population* or poorer world or developing econom* or less developed econom* or underdeveloped econom* or under developed econom* or middle income econom* or low income econom* or lower income econom* or low gdp or low gnp or low gross domestic or low gross national or lower gdp or lower gnp or lower gross domestic or lower gross national or lmic or lmics or third world or lami countr* or transitional countr* or emerging econom* or emerging nation*).mp.

5 Photovoltaic cells.mp.

6 Solar energy/

7 Renewable energy/

8 (Solar not Solar keratosis not Solar dermatitis not Solar lentigines not Solar urticaria not Solar plexus).mp. [mp=title, abstract, heading word, drug trade name, original title, device manufacturer, drug manufacturer, device trade name, keyword heading word, floating subheading word, candidate term word]

9 Electrification.mp.

10 Clean energy.mp.

11 Developing countries/ or low income countries/

12 (((lower income countries or low) and middle income countries) or LMIC* or low resource or global south).mp.

13 1 or 2 or 5 or 6 or 7 or 8 or 9 or 10

14 3 or 4 or 11 or 12

15 Hospitals/

16 Clinic*.mp.

17 Healthcare facilities/ 1

18 15 or 16 or 17

19 13 and 14 and 18

**Supplementary S5: SCOPUS Search Strategy**

Date: 10^th^ October 2024

( ALL ( ( ( "Hospitals" ) OR ( "Health Facilities" ) OR ( clinic ) OR ( hospitals ) ) ) AND ALL ( ( "Solar Energy" ) OR ( "Solar Power" ) OR ( "Solar Panel" ) OR ( "Photovoltaic" ) OR ( "Solar Cell*" ) ) AND ALL ( ( "Developing Countries" ) OR ( "developing countr*" ) OR ( "developing nation*" ) OR ( "developing world" ) OR ( "less developed countr*" ) OR ( "less developed nation*" ) OR ( "less developed world" ) OR ( "lesser developed countr*" ) OR ( "lesser developed nation*" ) OR ( "under developed countr*" ) OR ( "under developed nation*" ) OR ( "under developed world" ) OR ( "underdeveloped countr*" ) OR ( "underdeveloped nation*" ) OR ( "underdeveloped world" ) OR ( "middle income countr*" ) OR ( "middle income nation*" ) OR ( "middle income population*" ) OR ( "low income countr*" ) OR ( "low income nation*" ) OR ( "low income population*" ) OR ( "lower income countr*" ) OR ( "lower income nation*" ) OR ( "lower income population*" ) OR ( "underserved countr*" ) OR ( "underserved population*" ) OR ( "under served population*" ) OR ( "under served nation*" ) OR ( "under served population*" ) OR ( "deprived countr*" ) OR ( "deprived population*" ) OR ( "high burden countr*" ) OR ( "high burden nation*" ) OR ( "countdown countr*" ) OR ( "poor countr*" ) OR ( "poor nation*" ) OR ( "poor population*" ) OR ( "poor world" ) OR ( "poorer countr*" ) OR ( "poorer nation*" ) OR ( "poorer population*" ) OR ( "developing econom*" ) OR ( "less developed econom*" ) OR ( "underdeveloped econom*" ) OR ( "under developed econom*" ) OR ( "middle income econom*" ) OR ( "low income econom*" ) OR ( "lower income econom*" ) OR ( "low gdp" ) OR ( "low gnp" ) OR ( "low gross domestic" ) OR ( "low gross national" ) OR ( "lower gdp" ) OR ( "lower gross domestic" ) OR ( "lmic" ) OR ( "lmics" ) OR ( "third world" ) OR ( "lami countr*" ) OR ( "transitional countr*" ) OR ( "emerging econom*" ) OR ( "emerging nation*" ) ) )

**Supplementary S6: OVID Search Strategy**

Date: 10^th^ October 2024

1. Solar power.mp.

2. Solar technology.mp.

3. Photovoltaic cells.mp.

4. Solar energy/ (MeSH term for Solar Energy)

5. Renewable energy/ (MeSH term for Renewable Energy)

6. Solar.mp.

7. Electrification.mp.

8. Clean energy.mp.

9. 1 OR 2 OR 3 OR 4 OR 5 OR 6 OR 7 OR 8

10. Developing countries/ OR low income countries/ (MeSH terms)

11. (lower income countries OR low and middle income countries OR LMIC* OR low resource OR global south).mp.

12. (afghan* OR africa* OR albania* OR algeria* OR angola* OR argentin* OR armenia* OR azerbaijan* OR bangladesh* OR bengal* OR bangal* OR belarus* OR belorus* OR byelarus* OR byelorus* OR belize* OR benin* OR dahomey OR bhutan* OR bolivia* OR bosnia* OR herzegovin* OR botswan* OR batswan* OR bechuanaland* OR brazil* OR brasil* OR bulgaria* OR burkina* OR burkinese* OR upper volta* OR burundi* OR urundi* OR cabo verde* OR cape verde* OR cambodia* OR kampuchea* OR khmer* OR cameroon* OR cameroun* OR ubangi shari* OR chad* OR colombia* OR comoro* OR comore* OR comorian* OR mayotte* OR congo* OR zaire* OR costa rica* OR "cote d'ivoir*" OR "cote d' ivoir*" OR cote divoir* OR cote d ivoir* OR ivory coast* OR ivorian* OR cuba OR cuban OR cubans OR "cuba's" OR djibouti* OR french somaliland* OR dominica* OR ecuador* OR egypt* OR united arab republic* OR el salvador* OR salvadoran* OR guinea* OR equatoguinea* OR eritrea* OR eswatini* OR swaziland* OR swazi* OR swati* OR ethiopia* OR fiji* OR gabon* OR gabonese* OR gabonaise* OR gambia* OR ((georgia OR georgian OR georgians) NOT (atlanta OR california OR florida)) OR ghana* OR grenada* OR grenadian* OR guatemala* OR guyana* OR guiana* OR guyanese* OR haiti* OR hispaniola* OR hondura* OR india* OR indonesia* OR iran* OR iraq* OR jamaica* OR jordan* OR kazakh* OR kenya* OR karabati* OR korea* OR kosovo* OR kosova* OR kyrgyz* OR kirgiz* OR kirghiz* OR laos OR lao OR laotian* OR lebanon* OR lebanese* OR lesotho* OR lesothan* OR lesothonian* OR basutoland* OR mosotho* OR basotho* OR liberia* OR libya* OR jamahiriya* OR macedonia* OR madagasca* OR malagasy* OR malawi* OR nyasaland* OR malaysia* OR malay* federation OR maldives* OR maldivian* OR indian ocean OR mali OR malian* OR "mali's" OR micronesia* OR marshallese* OR kiribati* OR marshall island* OR tuvalu* OR mauritania* OR mauritan* OR mauritius* OR mexico* OR mexican* OR moldova* OR moldovia* OR mongol* OR montenegr* OR morocco* OR moroccan* OR ifni OR mozambique* OR mozambican* OR myanmar* OR burma* OR burmese OR namibia* OR nepal* OR netherlands antill* OR nicaragua* OR niger* OR omani OR omanis OR "oman's" OR pakistan* OR palestin* OR gaza* OR west bank* OR panama* OR paraguay* OR peru OR peruvian* OR "peru's" OR philippine* OR philipine* OR phillipine* OR phillippine* OR filipino* OR filipina* OR rwanda* OR rwandese OR ruanda* OR ruandese OR samoa* OR navigator island* OR pacific island* OR polynesia* OR "sao tome and principe*" OR sao tomean* OR santomean* OR senegal* OR serbia* OR sierra leone* OR melanesia* OR solomon island* OR norfolk island* OR somali* OR sri lanka* OR ceylon* OR saint lucia* OR st lucia* OR saint vincent* OR st vincent* OR vincentian* OR grenadine* OR sudan* OR surinam* OR syria* OR tajik* OR tadjik* OR tadzhik* OR tanzania* OR tanganyika* OR thai* OR timor leste* OR east timor* OR timorese* OR togo OR togoles* OR "togo's" OR tonga* OR tunisia* OR turkiy* OR turkey* OR turk OR turks OR turkish OR turkmen* OR uganda* OR ukrain* OR uzbek* OR vanuatu* OR new hebrides* OR venezuela* OR vietnam* OR viet nam* OR yemen* OR yugoslav* OR zambia* OR zimbabwe* OR rhodesia* OR arab* countr* OR middle east* OR global south OR sahara* OR subsahara* OR magreb* OR maghrib* OR west indies* OR caribbean* OR central america* OR latin america* OR south america* OR central asia* OR north asia* OR northern asia* OR southeastern asia* OR south eastern asia* OR southeast asia* OR south east asia* OR west asia* OR western asia* OR east europe* OR eastern europe*).mp.

13. ({Moner-Girona, 2021 #817}mp.

14. 10 OR 11 OR 12 OR 13

15. Hospitals/ (MeSH term for hospitals)

16. Clinic*.mp.

17. Healthcare facilities/ (MeSH term for Healthcare Facilities)

18. 15 OR 16 OR 17

19. 9 AND 14 AND 18

**Supplemental S7: Cochrane Search Strategy**

Date Run: 11/10/2024 20:09:44

#1 (Solar power):ti,ab,kw (Word variations have been searched)

#2 MeSH descriptor: [Solar Energy] explode all trees

#3 Solar technology

#4 MeSH descriptor: [Renewable Energy] explode all trees

#5 Solar

#6 Electrification

#7 Clean energy

#8 Photovoltaic

#9 #1 OR #2 OR #3 OR #4 OR #5 OR #6 OR #7 OR #8

#10 MeSH descriptor: [Developing Countries] explode all trees

#11 lower income countries OR low and middle income countries OR LMIC* OR low resource OR global south

#12 afghan* OR africa* OR albania* OR algeria* OR angola* OR argentin* OR armenia* OR azerbaijan* OR bangladesh* OR bengal* OR bangal* OR belarus* OR belorus* OR byelarus* OR byelorus* OR belize* OR benin* OR dahomey OR bhutan* OR bolivia* OR bosnia* OR herzegovin* OR botswan* OR batswan* OR bechuanaland* OR brazil* OR brasil* OR bulgaria* OR burkina* OR burkinese* OR upper volta* OR burundi* OR urundi* OR cabo verde* OR cape verde* OR cambodia* OR kampuchea* OR khmer* OR cameroon* OR cameroun* OR ubangi shari* OR chad* OR colombia* OR comoro* OR comore* OR comorian* OR mayotte* OR congo* OR zaire* OR costa rica* OR "cote d'ivoir*" OR "cote d' ivoir*" OR cote divoir* OR cote d ivoir* OR ivory coast* OR ivorian* OR cuba OR cuban OR cubans OR "cuba's" OR djibouti* OR french somaliland* OR dominica* OR ecuador* OR egypt* OR united arab republic* OR el salvador* OR salvadoran* OR guinea* OR equatoguinea* OR eritrea* OR eswatini* OR swaziland* OR swazi* OR swati* OR ethiopia* OR fiji* OR gabon* OR gabonese* OR gabonaise* OR gambia* OR ((georgia OR georgian OR georgians) NOT (atlanta OR california OR florida)) OR ghana* OR grenada* OR grenadian* OR guatemala* OR guyana* OR guiana* OR guyanese* OR haiti* OR hispaniola* OR hondura* OR india* OR indonesia* OR iran* OR iraq* OR jamaica* OR jordan* OR kazakh* OR kenya* OR karabati* OR korea* OR kosovo* OR kosova* OR kyrgyz* OR kirgiz* OR kirghiz* OR laos OR lao OR laotian* OR lebanon* OR lebanese* OR lesotho* OR lesothan* OR lesothonian* OR basutoland* OR mosotho* OR basotho* OR liberia* OR libya* OR jamahiriya* OR macedonia* OR madagasca* OR malagasy* OR malawi* OR nyasaland* OR malaysia* OR malay* federation OR maldives* OR maldivian* OR indian ocean OR mali OR malian* OR "mali's" OR micronesia* OR marshallese* OR kiribati* OR marshall island* OR tuvalu* OR mauritania* OR mauritan* OR mauritius* OR mexico* OR mexican* OR moldova* OR moldovia* OR mongol* OR montenegr* OR morocco* OR moroccan* OR ifni OR mozambique* OR mozambican* OR myanmar* OR burma* OR burmese OR namibia* OR nepal* OR netherlands antill* OR nicaragua* OR niger* OR omani OR omanis OR "oman's" OR pakistan* OR palestin* OR gaza* OR west bank* OR panama* OR paraguay* OR peru OR peruvian* OR "peru's" OR philippine* OR philipine* OR phillipine* OR phillippine* OR filipino* OR filipina* OR rwanda* OR rwandese OR ruanda* OR ruandese OR samoa* OR navigator island* OR pacific island* OR polynesia* OR "sao tome and principe*" OR sao tomean* OR santomean* OR senegal* OR serbia* OR sierra leone* OR melanesia* OR solomon island* OR norfolk island* OR somali* OR sri lanka* OR ceylon* OR saint lucia* OR st lucia* OR saint vincent* OR st vincent* OR vincentian* OR grenadine* OR sudan* OR surinam* OR syria* OR tajik* OR tadjik* OR tadzhik* OR tanzania* OR tanganyika* OR thai* OR timor leste* OR east timor* OR timorese* OR togo OR togoles* OR "togo's" OR tonga* OR tunisia* OR turkiy* OR turkey* OR turk OR turks OR turkish OR turkmen* OR uganda* OR ukrain* OR uzbek* OR vanuatu* OR new hebrides* OR venezuela* OR vietnam* OR viet nam* OR yemen* OR yugoslav* OR zambia* OR zimbabwe* OR rhodesia* OR arab* countr* OR middle east* OR global south OR sahara* OR subsahara* OR magreb* OR maghrib* OR west indies* OR caribbean* OR central america* OR latin america* OR south america* OR central asia* OR north asia* OR northern asia* OR southeastern asia* OR south eastern asia* OR southeast asia* OR south east asia* OR west asia* OR western asia* OR east europe* OR eastern europe*

#13 developing countr* OR developing nation* OR developing population* OR developing world OR less developed countr* OR less developed nation* OR less developed world OR lesser developed countr* OR lesser developed nation* OR lesser developed world OR under developed countr* OR under developed nation* OR under developed world OR underdeveloped countr* OR underdeveloped nation* OR underdeveloped world OR middle income countr* OR middle income nation* OR middle income population* OR low income countr* OR low income nation* OR low income population* OR lower income countr* OR lower income nation* OR lower income population* OR underserved countr* OR underserved nation* OR underserved population* OR under served population* OR under served nation* OR under served population* OR deprived countr* OR deprived population* OR high burden countr* OR high burden nation* OR countdown countr* OR countdown nation* OR poor countr* OR poor nation* OR poor population* OR poor world OR poorer countr* OR poorer nation* OR poorer population* OR poorer world OR developing econom* OR less developed econom* OR underdeveloped econom* OR under developed econom* OR middle income econom* OR low income econom* OR lower income econom* OR low gdp OR low gnp OR low gross domestic OR low gross national OR lower gdp OR lower gnp OR lower gross domestic OR lower gross national OR lmic OR lmics OR third world OR lami countr* OR transitional countr* OR emerging econom* OR emerging nation*

#14 #10 OR #11 OR #12 OR #13

#15 MeSH descriptor: [Hospitals] explode all trees

#16 Clinic

#17 MeSH descriptor: [Health Facilities] explode all trees

#18 #15 OR #16 OR #17

#19 #9 AND #14 AND #18

#20 sodis

**Supplemental S8: Web of Science Search Strategy**

Date: 10^th^ October 2024

hospital* OR "health facilit*" OR clinic* (All Fields) AND "solar energy" OR "solar power" OR "solar panel*" OR photovoltaic* OR "solar cell*" (All Fields) AND "developing countr*" OR "developing nation*" OR "developing world" OR "less developed countr*" OR "less developed nation*" OR "less developed world" OR "under developed countr*" OR "under developed nation*" OR "under developed world" OR underdeveloped countr* OR underdeveloped nation* OR underdeveloped world OR "middle income countr*" OR "middle income nation*" OR "middle income population*" OR "low income countr*" OR "low income nation*" OR "low income population*" OR "lower income countr*" OR "lower income nation*" OR "lower income population*" OR underserved countr* OR "underserved population*" OR "under served population*" OR deprived countr* OR "deprived population*" OR "high burden countr*" OR "high burden nation*" OR "poor countr*" OR "poor nation*" OR "poor population*" OR "poor world" OR "developing econom*" OR "emerging econom*" OR "emerging nation*" OR lmic OR lmics OR "low and middle income countr*" OR "low resource" OR "global south" OR "third world" OR "lower income countries" OR "low gdp" OR "low gnp" (All Fields)

**Supplemental S9: JSTOR Search Strategy**

(((("developing countr*" OR "low income countr*" OR "middle income countr*" OR "low and middle income countr*" OR LMIC* OR "global south")) AND (hospital* OR clinic* OR "health facilit*")) AND ("solar energy" OR "solar power" OR "solar panel*" OR photovoltaic* OR "solar cell*"))

**Supplementary S10: Healthcare facility solar installation case studies:**

These three case studies are real world examples of implementing solar power in low- and middle-income country hospitals. For all three hospitals, installation costs were covered by international grants and philanthropic support—funding pathways that remain out of reach for the vast majority of hospitals globally.

1. **Tertiary hospital in Sub-Saharan Africa**

This hospital receives its main electricity from the electrical grid, although power-outages occur 5-6 times per day. The hospital does have a back-up diesel generator, although this normally takes minimum 5 minutes to manually switch on during outages and emits heavy pollutants, both contributing to global warming and impacting local air quality. The generator requires frequent maintenance and cooling off intervals, meaning that there can be extended periods without electricity. This regularly causes delays or cancellations to surgery, interruptions in the laboratory, and disruptions to patient care.

The aim of the project was to fully electrify one building, containing critical care and operating theatres, with solar power and battery systems. The estimated load of the unit was 30KW.

The high upfront cost of solar power installation was covered by a research grant from an international organisation. Cooperation between international organisations was essential for the planning and delivery of this project. One of the main challenges was uncertainty about future hospital planning; the hospital had provisional plans to build on the existing roof space. There was delay in the project while the hospital team confirmed that the roof could be used for the solar array.

Additional challenges included inconsistent and unreliable quotes from local electricity companies, which lacked due diligence. Additionally, maintenance contracts were very short, with most companies offering less than 5 years. However, through international collaborations, an international charity with experience in implementing hospital solar power in Africa became involved in the project. They were able to identify local suppliers where due diligence has been established.

A 40KW Solar power system was installed in September 2025, with five inverters and a 70KWh battery back-up system. This was a hybrid system, allowing operation with the electrical grid and diesel generator.

Following installation, there were no further power outages for several months, as the system worked well. This allowed surgery to proceed uninterrupted by power outages, improving efficiency and patient care. However, after 3 months, the solar system failed, requiring the inverters to be replaced. There was significant delay in replacement of the inverters, meaning the operating theatres and critical care once again had regular interruptions by power outages.

1. **Not for Profit referral Hospital in Sub-Saharan Africa**

Power interruptions are a daily reality at this 250-bed referral hospital in a remote region of sub-Saharan Africa, averaging over 1.5 hours of outage each day. These disruptions have directly contributed to patient mortality through the failure of critical equipment such as oxygen concentrators, incubators and baby warmers. Previous efforts to implement a decentralised hydroelectric system, drawing from a nearby river, proved unfeasible due to prohibitive upfront costs and complex engineering requirements.

In response, a UK based steering group pivoted to on-site solar energy. Funding was obtained through philanthropic activities in the UK. However, there was insufficient capital for battery storage, requiring a more bespoke solar system and introducing significant design challenges. Extensive collaboration between UK-based and local engineers was required, incurring both logistical delays and consulting fees.

The hospital's remote location additionally meant that regular maintenance contracts could not be established, as it was expensive and time-consuming for skilled technicians to reach the site. To mitigate against operation failure in the event of an inverter breaking, microinverters will likely be used. Trust and capacity assessment of local suppliers and technicians also posed difficulties.

The primary barrier in this case-study was lack of funding for a standard solar and battery system array, despite international philanthropic funding. This project demonstrates how international cooperation was essential in addressing energy insecurity in low-resource health settings. Engineers from high-income countries (HICs) voluntarily contributed expertise to validate and co-design the system. It also demonstrates the challenges of designing bespoke energy solutions. Future projects would benefit from incorporating battery storage and adopting standardised, modular solar solutions to enhance reliability and ease of maintenance.

1. **Secondary Hospital in Central India**

This remote hospital in India has multiple power outages per day, during which operations are cancelled, critical care ventilators fail and the radiology department are unable to perform scans. A week-long survey demonstrated multiple disruptions and cancellations to surgery due to power outages, with equipment additionally failing following voltage surges from the electrical grid. The hospital has high monthly costs for electricity from the grid and diesel generators.

The aim of the project was to electrify the entire hospital with solar energy and battery systems. This was conducted using local suppliers and installers, led by the local hospital leaders with support from an international steering group.

This project was ultimately unsuccessful. Due to the remote location of the hospital, it was challenging for suppliers to visit and provide estimates. Additionally, there was a lack of consistency and accountability from local business. Although several quotes were received, they differed drastically in energy needs assessments, equipment requirements and costs.
