# Supplementary material for: Barriers, facilitators and strategies for implementing on-site hospital solar power in low- and middle-income countries: a systematic review, global prioritisation survey and development of an implementation tool
Source: BMJ Glob Health. 2026 Jun 18;11(6):e023926. doi: 10.1136/bmjgh-2026-023926 (PMC13288949; doi:10.1136/bmjgh-2026-023926)

Supplementary Table S1: Inclusion and exclusion criteria

|  | Inclusion Criteria | Exclusion Criteria |
| --- | --- | --- |
| Type of study | Published quantitative, qualitative and mixed method studies, letters, literature reviews and reports | Abstracts, protocols/registered studies, older than 15 years |
| Setting | LMICs, on-site PV implementation in hospitals, healthcare facilities or primary care facilities | Non-healthcare settings  Population health studies  Specialised PV powered medical devices, high income countries |

Supplementary Table S2: Search Terms

| Population | lower income countries, low and middle income countries (MeSH), developing countries (MeSH), LMICs, low resource, global south |
| --- | --- |
| Intervention | solar, solar power, solar energy (MeSH), solar technology, photovoltaic cells, renewable energy (Mesh), electrification, clean energy |
| Setting | hospitals (MeSH), clinic, healthcare facilities (MeSH) |

Supplementary Table 3: Papers included with extracted barrier and facilitator themes (pre-synthesis)

| *Paper* | *Author* | *Year* | *Barrier* | *Facilitator* |
| --- | --- | --- | --- | --- |
| *A review of sustainable energy access and technologies for healthcare facilities in the Global South* | Franco et al. | 2017 | Emissions from manufacturing and disposal | Adapt medical technology to run on DC |
|  |  |  | Lack of local suppliers | Battery cycling and combination |
|  |  |  | High upfront and maintenance costs | Consider hybrid energy system |
|  |  |  | Irregular power supply | Generate local business and markets |
|  |  |  | Limit to service delivery availability | Improve equipment and HVAC efficiency |
|  |  |  | Low cost of diesel generator | Multi-national and organisational engagement and partnership |
|  |  |  | Needs complex energy assessment | New & cheaper technologies |
|  |  |  | Needs other energy sources/battery | Prioritise critical loads |
|  |  |  | Not easily moved/changed | Recycling plan |
|  |  |  | Not tailored to local community |  |
|  |  |  | PV damaged by dust/temperatures/humidity/rain | |
|  |  |  | PV damaged by earthquake | |
|  |  |  | Requires DC to AC converter | |
|  |  |  | Requires regular maintenance | |
|  |  |  | Requires skilled workers | |
|  |  |  | Risk of theft | |
|  |  |  | Unfamiliar with PV | |
| *Access to Modern Energy Services for Health Facilities in Resource-Constrained Settings* | WHO | 2014 | Battery short life span/fail in hot climates | Adapt medical technology to run on DC |
|  |  |  | High upfront and maintenance costs | Assist with upfront costs and sell surplus energy |
|  |  |  | Emissions from manufacturing and disposal | Consider hybrid energy system |
|  |  |  | Not prioritised | Generate local business and markets |
|  |  |  | Requires skilled workers | Improve equipment and HVAC efficiency |
|  |  |  | Risk of theft | Market-oriented financing and remuneration |
|  |  |  |  | Repurposed money and grants |
|  |  |  |  | Subsidies for PV installation and companies |
| *Achieving universal electrification of rural healthcare facilities in sub-Saharan Africa with decentralized renewable energy technologies* | Moner-Girona et al. | 2021 | Lack of global data and research | New & cheaper technologies |
|  |  |  | Varying solar radiation/seasonality | Techno-economic analysis (HOMER) |
| *Advancing Solar Energy for Primary Healthcare in Developing Nations* | Sharma et al. | 2024 | Insufficient budget | Bilateral investment treaties |
|  |  |  | Irregular power supply | High quality equipment |
|  |  |  | Not prioritised | New & cheaper technologies |
|  |  |  | Poor handover from NGO to national partner | Philanthropic or private sector help with costs |
|  |  |  | Requires DC to AC converter | Prioritise critical loads |
|  |  |  | Requires regular maintenance | Prioritise transparent energy/healthcare policies |
|  |  |  |  | Strong advocacy for health/energy linkage |
| *An Overview of Energy Access Solutions for Rural Healthcare Facilities* | Olatomiwa et al. | 2022 | High upfront and maintenance costs | Battery cycling and combination |
|  |  |  | Insufficient budget | Consider hybrid energy system |
|  |  |  | Emissions from manufacturing and disposal | Continuous mobilization and consultation |
|  |  |  | Battery short life span/fail in hot climates | Dedicated maintenance funding |
|  |  |  | Irregular power supply | Demand side management |
|  |  |  | Lack of community contribution/engagement | Ensure maintenance contracts and funding |
|  |  |  | Requires regular maintenance | Market-oriented financing and remuneration |
|  |  |  | Requires skilled workers | National Research Funding |
|  |  |  |  | New & cheaper technologies |
|  |  |  |  | Prioritise transparent energy/healthcare policies |
|  |  |  |  | Public private partnership |
|  |  |  |  | Remote monitoring for maintenance |
|  |  |  |  | Community and Staff Ownership |
|  |  |  |  | Subsidies for PV installation and companies |
|  |  |  |  | Tax waivers for PV imports and industries |
|  |  |  |  | Techno-economic analysis (HOMER) |
|  |  |  |  | Track supportive policies |
| *Building integrated photovoltaic with hydrogen storage as a sustainable solution in Iranian rural healthcare centers* | Dehshiri et al. | 2024 | Inaccessible for installation | Bilateral investment treaties |
|  |  |  | Emissions from manufacturing and disposal | Consider hybrid energy system |
|  |  |  | Battery short life span/fail in hot climates | New & cheaper technologies |
|  |  |  | Lack of global data and research | Techno-economic analysis (HOMER) |
|  |  |  | Lack of generalisable global research | |
|  |  |  | PV damaged by dust/temperatures/humidity/rain | |
|  |  |  | Varying solar radiation/seasonality | |
| *Challenges of Implementing Antenatal Ultrasound Screening in a Rural Study Site: A Case Study From the Democratic Republic of the Congo* | Swanson et al. | 2017 | Building design prevents installation | Alternate building solutions |
|  |  |  | Risk of theft | Locked boxes |
|  |  |  |  | Security personnel |
| *Challenges of Phasing out Emergency Diesel Generators: The Case Study of Lacor Hospital's Energy Community* | Felice et al. | 2023 | High upfront and maintenance costs | Public private partnership |
|  |  |  | Lack of local training | Community and Staff Ownership |
|  |  |  | Requires skilled workers | |
|  |  |  | Unable to use additional power due to unstable grid | |
| *Electricity access, community healthcare service delivery, and rural development nexus: Analysis of 3 solar electrified CHPS in off‐grid communities in Ghana* | Opoku et al. | 2020 | Unable to access existing finance | Assist with upfront costs and sell surplus energy |
|  |  |  |  | Consider hybrid energy system |
|  |  |  |  | Ensure maintenance contracts and funding |
|  |  |  |  | Market-oriented financing and remuneration |
|  |  |  |  | Plan for future energy needs with overhead |
|  |  |  |  | Strong advocacy for health/energy linkage |
| *Electrification Planning for Healthcare Facilities in Low-Income Countries, Application of a Portfolio-Level, Multi Criteria Decision-Making Approach* | Pakravan et al. | 2021 | Inaccessible for installation | Community capacity building |
|  |  |  | Battery short life span/fail in hot climates | Continuous geospatial data platforms |
|  |  |  | Irregular power supply | Multi-criteria decision tools to prioritise sites |
|  |  |  | No standards for design and implementation | Remote monitoring for maintenance |
|  |  |  | Not tailored to local community | Robust O&M plan |
|  |  |  | Poor allocation of resources | Community and Staff Ownership |
|  |  |  | Requires regular maintenance | Techno-economic analysis (HOMER) |
|  |  |  | Requires skilled workers | |
| *Energizing health: accelerating electricity access in health-care facilities* | WHO | 2023 | Emissions from manufacturing and disposal | Community and staff training |
|  |  |  | Insufficient budget | Community capacity building |
|  |  |  | Lack of local suppliers | Continuous geospatial data platforms |
|  |  |  | High upfront and maintenance costs | Dedicated maintenance funding |
|  |  |  | Investment Accountability | Demand side management |
|  |  |  | Lack of global data and research | Ensure maintenance contracts and funding |
|  |  |  | Lack of coordination between health and energy sectors | Focused advocacy for PV in government |
|  |  |  | Lack of coordination with medical device procurement | Health facility management groups and champions |
|  |  |  | Limit to service delivery availability | Improve equipment and HVAC efficiency |
|  |  |  | No standards for design and implementation | Long term monitoring |
|  |  |  | No supportive policies | Multi-national and organisational engagement and partnership |
|  |  |  | Not tailored to local community | Philanthropic or private sector help with costs |
|  |  |  | Obstructive Regulations | Prioritise transparent energy/healthcare policies |
|  |  |  | Requires skilled workers | Public call in local newspapers/media |
|  |  |  |  | Public private partnership |
|  |  |  |  | Remote and on-site needs assessment |
|  |  |  |  | Remote monitoring for maintenance |
|  |  |  |  | Community and Staff Ownership |
|  |  |  |  | Strong advocacy for health/energy linkage |
|  |  |  |  | Tax waivers for PV imports and industries |
|  |  |  |  | Techno-economic analysis (HOMER) |
| *Energy access in Malawian healthcare facilities: consequences for health service delivery and environmental health conditions* | Reuland et al. | 2019 | Delay in maintenance | Philanthropic or private sector help with costs |
|  |  |  | Lack of local suppliers | |
|  |  |  | Insufficient budget | |
|  |  |  | Limit to service delivery availability | |
|  |  |  | Not prioritised | |
|  |  |  | Requires skilled workers | |
|  |  |  | Varying solar radiation/seasonality | |
| *Energy consumption profile estimation and benefits of hybrid solar energy system adoption for rural health units in the Philippines* | Lemence et al. | 2021 | Battery short life span/fail in hot climates | Techno-economic analysis (HOMER) |
|  |  |  | Needs complex energy assessment | |
|  |  |  | Varying solar radiation/seasonality | |
| *Evaluating the impact of adding energy storage on the performance of a hybrid power system* | Jacobus et al. | 2011 | Irregular power supply | Assist with upfront costs and sell surplus energy |
|  |  |  | Needs complex energy assessment | Consider hybrid energy system |
|  |  |  |  | Demand side management |
|  |  |  |  | Remote and on-site needs assessment |
| *Evaluation of a grid-independent solar photovoltaic system for primary health centres (PHCs) in developing countries* | Babatunde et al. | 2018 |  | Clear regulations and guidelines |
|  |  |  |  | Countries have raw resources |
|  |  |  |  | Demand side management |
|  |  |  |  | Techno-economic analysis (HOMER) |
| *Green electricity and medical electrolytic oxygen from solar energy-A sustainable solution for rural hospitals* | Ngoh et al. | 2022 |  | New & cheaper technologies |
| *Health and Energy Platform of Action report 2020-2022: building connections for better health* | WHO | 2024 | Lack of coordination between health and energy sectors | Focused advocacy for PV in government |
|  |  |  |  | Focused advocacy for PV in government |
|  |  |  |  | Community and staff training |
|  |  |  |  | Focused advocacy for PV in government |
|  |  |  |  | Public-private-partnership delivery forum |
|  |  |  |  | Multistakeholder meetings, side events |
|  |  |  |  | Multi-national and organisational engagement and partnership |
|  |  |  |  | Multistakeholder meetings, side events |
|  |  |  |  | Strong advocacy for health/energy linkage |
|  |  |  |  | Multi-national and organisational engagement and partnership |
|  |  |  |  | Market-oriented financing and remuneration |
|  |  |  |  | Repurposed money and grants |
|  |  |  |  | Community capacity building |
|  |  |  |  | Demand side management |
|  |  |  |  | Comprehensive short- and long-term roadmap |
|  |  |  |  | Multi-national and organisational engagement and partnership |
|  |  |  |  | Multi-national and organisational engagement and partnership |
|  |  |  |  | Online workshops, videos and communication |
|  |  |  |  | Online workshops, videos and communication |
|  |  |  |  | Online workshops, videos and communication |
| *Implementation of a 3-phase grid-coupled solar electricity and back-up system at Mulanje Mission Hospital, Malawi* | Glas et al. | 2024 | Lack of local suppliers | Adapt medical technology to run on DC |
|  |  |  | High upfront and maintenance costs | Assist with upfront costs and sell surplus energy |
|  |  |  | Insufficient budget | Battery cycling and combination |
|  |  |  | Irregular power supply | Community and staff training |
|  |  |  | Lack of local training | Consider hybrid energy system |
|  |  |  | Needs complex energy assessment | Control system with mobile app |
|  |  |  | Not prioritised | Demand side management |
|  |  |  | Unable to use additional power due to unstable grid | Ensure maintenance contracts and funding |
|  |  |  |  | High quality equipment |
|  |  |  |  | Prioritise critical loads |
|  |  |  |  | Reuse existing equipment |
| *Implementation research on sustainable electrification of rural primary care facilities in Ghana and Uganda* | Javadi et al. | 2020 | Delay in maintenance | Adapt and engage in local solutions |
|  |  |  | High turnover hospital staff | Community and staff training |
|  |  |  | Insufficient budget | Community capacity building |
|  |  |  | Lack of community contribution/engagement | Continuous mobilization and consultation |
|  |  |  | Lack of coordination with medical device procurement | Demand side management |
|  |  |  | Lack of local training | Ensure maintenance contracts and funding |
|  |  |  | Limit to service delivery availability | Establish workflow between changing staff |
|  |  |  | Requires skilled workers | Health facility management groups and champions |
|  |  |  | Risk of theft | Long term monitoring |
|  |  |  |  | Meet local expectations and maintain trust |
|  |  |  |  | Multi-criteria decision tools to prioritise sites |
|  |  |  |  | Multi-national and organisational engagement and partnership |
|  |  |  |  | Multistakeholder meetings, side events |
|  |  |  |  | Plan for future energy needs with overhead |
|  |  |  |  | Remote and on-site needs assessment |
|  |  |  |  | Sensitizing district leaders and health workers |
|  |  |  |  | Community and Staff Ownership |
|  |  |  |  | Strong advocacy for health/energy linkage |
|  |  |  |  | Targeted and asset-based strategies |
| *Powering Rural Healthcare with Sustainable Energy: A Global Review of Solar Solutions* | Izuka et al. | 2023 | Inaccessible for installation | Clear regulations and guidelines |
|  |  |  | High upfront and maintenance costs | Community and staff training |
|  |  |  | Lack of healthcare impact research | Public private partnership |
|  |  |  | Lack of local training | |
|  |  |  | No supportive policies | |
|  |  |  | PV damaged by dust/temperatures/humidity/rain | |
|  |  |  | Requires skilled workers | |
| *Regional hospitals in humid tropical climate - Guidelines for sustainable design* | Ignjatovic et al. | 2018 | PV damaged by dust/temperatures/humidity/rain | Improve equipment and HVAC efficiency |
|  |  |  |  | Technologies to mitigate PV damage |
| *Rural healthcare workers views on the introduction of solar power and oxygen concentrators in health facilities in Papua New Guinea: a qualitative study* | Pulsan et al. | 2021 | Requires regular maintenance | |
| *Solar Energy Implementation for Health-Care Facilities in Developing and Underdeveloped Countries: Overview, Opportunities, and Challenges* | Soto et al. | 2022 | Emissions from manufacturing and disposal | |
|  |  |  | High land usage | |
|  |  |  | High upfront and maintenance costs | |
|  |  |  | Investment Accountability | |
|  |  |  | Limit to service delivery availability | |
|  |  |  | No supportive policies | |
|  |  |  | Not prioritised | |
|  |  |  | Poor handover from NGO to national partner | |
|  |  |  | Requires skilled workers | |
|  |  |  | Varying solar radiation/seasonality | |
| *Solar energy: A panacea for the electricity generation crisis in Nigeria* | Agbo et al. | 2021 | Lack of local suppliers | Adapt and engage in local solutions |
|  |  |  | High upfront and maintenance costs | Bilateral investment treaties |
|  |  |  | Lack of community contribution/engagement | Clear regulations and guidelines |
|  |  |  | Not prioritised | Community and staff training |
|  |  |  | Poor allocation of resources | Countries have raw resources |
|  |  |  |  | Declining alternative compliance payment rates |
|  |  |  |  | High solar iridescence |
|  |  |  |  | Market-oriented financing and remuneration |
|  |  |  |  | National Research Funding |
|  |  |  |  | Prioritise transparent energy/healthcare policies |
|  |  |  |  | Public call in local newspapers/media |
|  |  |  |  | Set appropriate capacity limits |
|  |  |  |  | Subsidies for PV installation and companies |
|  |  |  |  | Technologies to mitigate PV damage |
| *Solar panels bring power to rural health facilities* | WHO | 2023 | High upfront and maintenance costs | New & cheaper technologies |
| *Solar Power for Resilient Health Systems in Nigeria: Regulatory, Financial and Organisational Options for Sustainable Business Models* | Paim et al. | 2022 | High upfront and maintenance costs | Assist with upfront costs and sell surplus energy |
|  |  |  | Insufficient budget | Bilateral investment treaties |
|  |  |  | Lack of coordination between health and energy sectors | Generate local business and markets |
|  |  |  | Lack of coordination with medical device procurement | Multi-criteria decision tools to prioritise sites |
|  |  |  | Obstructive Regulations | Philanthropic or private sector help with costs |
|  |  |  | Poor allocation of resources | Public private partnership |
|  |  |  | Poor handover from NGO to national partner | Robust framework to help banks invest in energy |
|  |  |  | Requires skilled workers | Strong advocacy for health/energy linkage |
|  |  |  |  | Subsidies for PV installation and companies |
|  |  |  |  | Tax waivers for PV imports and industries |
| *Solar powered oxygen systems in remote health centers in Papua New Guinea: a large scale implementation effectiveness trial* | Duke et al. | 2017 | Lack of global data and research | Continuous mobilization and consultation |
|  |  |  | No supportive policies | Ensure maintenance contracts and funding |
|  |  |  | Not prioritised | High quality equipment |
|  |  |  |  | Long term monitoring |
|  |  |  |  | Multi-criteria decision tools to prioritise sites |
|  |  |  |  | Remote and on-site needs assessment |
|  |  |  |  | Strong advocacy for health/energy linkage |
| *Solar powered oxygen systems in remote health centers in Papua New Guinea: a large scale implementation effectiveness trial* | Duke et al. | 2017 | High turnover hospital staff | |

Supplementary Table S4: The most referenced summarised barriers and facilitators to on-site hospital solar panel implementation from the systematic review

| **Barriers** | **Count** | **Facilitators** | **Count** |
| --- | --- | --- | --- |
| High upfront costs of solar panels | 12 | Community and Staff training for installation and maintenance | 7 |
| Requires skilled workers for installation and maintenance | 11 | Hybrid energy system (combining solar, electrical grid and generators) | 7 |
| Insufficient hospital budget for solar panels | 7 | Prioritise critical electrical loads during peak hours | 7 |
| Not government prioritisation | 7 | Multi-national and organisational engagement and partnership | 7 |
| Short battery life span | 6 | New & cheaper solar and battery technologies | 7 |
| Emissions from solar panel manufacturing and disposal | 6 | Strong government advocacy for health and energy partnerships | 7 |
| Solar panels provide irregular power supply | 6 | Techno-economic analysis (HOMER) | 7 |

Supplementary Table S5: Characteristics of survey respondents

|  |  | High | Upper middle | Lower middle | Low | Total |
| --- | --- | --- | --- | --- | --- | --- |
| Role | Doctor | 214 (96.8) | 89 (83.2) | 124 (93.9) | 19 (90.5) | 446 (92.7) |
|  | Other (Healthcare  professionals,  administration,  researchers) | 7 (3.2) | 18 (16.8) | 8 (6.1) | 2 (9.5) | 35 (7.3) |
| Hospital Type | Primary level | 12 (5.4) | 8 (7.5) | 9 (6.8) | 7 (33.3) | 36 (7.5) |
|  | Secondary level | 39 (17.6) | 16 (15.0) | 11 (8.3) | 7 (33.3) | 73 (15.2) |
|  | Tertiary level | 170 (76.9) | 83 (77.6) | 112 (84.8) | 7 (33.3) | 372 (77.3) |
| Hospital Funding | Corporate | 14 (6.3) | 14 (13.1) | 18 (13.6) | 4 (19.0) | 50 (10.4) |
|  | NGO | 9 (4.1) | 3 (2.8) | 7 (5.3) | 2 (9.5) | 21 (4.4) |
|  | National  Government | 112 (50.7) | 56 (52.3) | 73 (55.3) | 5 (23.8) | 246 (51.1) |
|  | State/Local  Government | 86 (38.9) | 34 (31.8) | 34 (25.8) | 10 (47.6) | 164 (34.1) |
| Solar panels Installed | No | 172 (77.8) | 95 (88.8) | 43 (32.6) | 16 (76.2) | 326 (67.8) |
|  | Yes | 49 (22.2) | 12 (11.2) | 89 (67.4) | 5 (23.8) | 155 (32.2) |
| Solar panels working | No | 2 (4.5) | 1 (8.3) | 3 (3.5) |  | 6 (4.1) |
|  | Yes | 42 (95.5) | 11 (91.7) | 83 (96.5) | 5 (100.0) | 141 (95.9) |
| Previous attempt to install solar | No | 167 (97.1) | 85 (89.5) | 37 (86.0) | 14 (87.5) | 303 (92.9) |
|  | Yes | 5 (2.9) | 10 (10.5) | 6 (14.0) | 2 (12.5) | 23 (7.1) |
| Solar panel installation planned | No | 42 (19.0) | 22 (20.6) | 17 (12.9) | 7 (33.3) | 88 (18.3) |
|  | Not sure | 111 (50.2) | 71 (66.4) | 54 (40.9) | 9 (42.9) | 245 (50.9) |
|  | Yes | 68 (30.8) | 14 (13.1) | 61 (46.2) | 5 (23.8) | 148 (30.8) |

Supplementary Figure S1: Heatmap of survey respondents across low- and middle-income countries


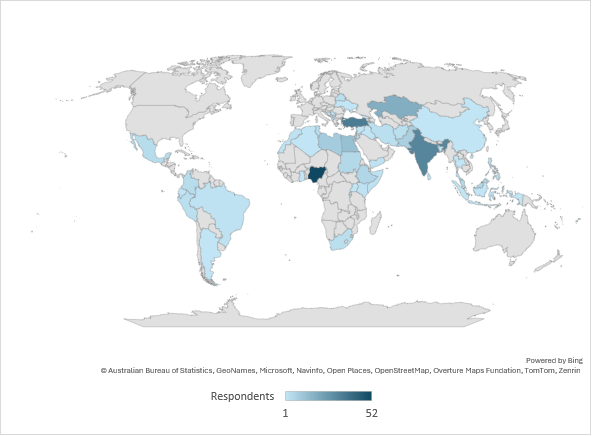


Supplementary Figure S2: Difference in prioritization of importance of A) barriers and B) facilitators between high income (HIC) and LMIC respondents, values are percentage differences. Implementation factors are grouped by the CFIR.


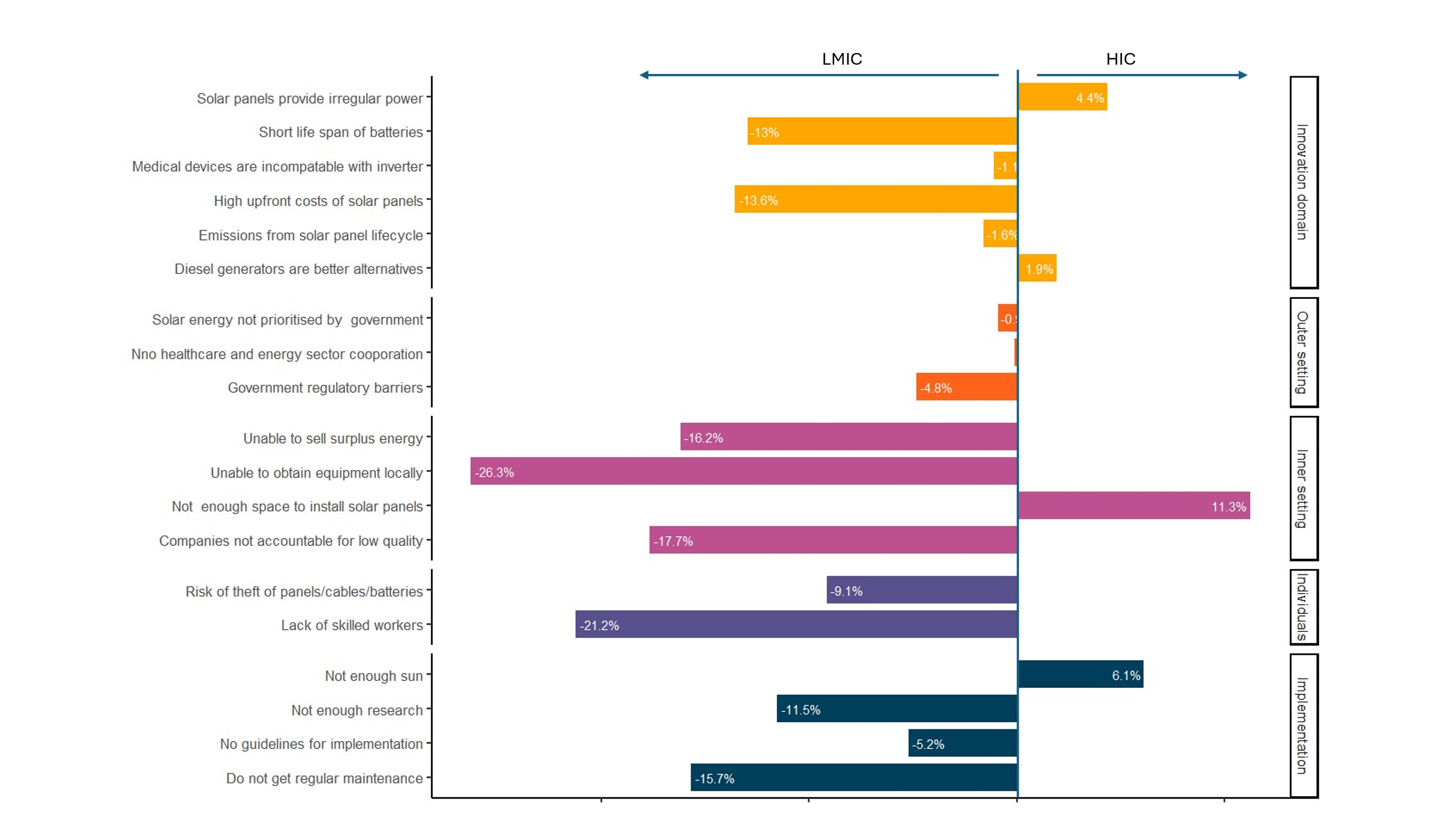

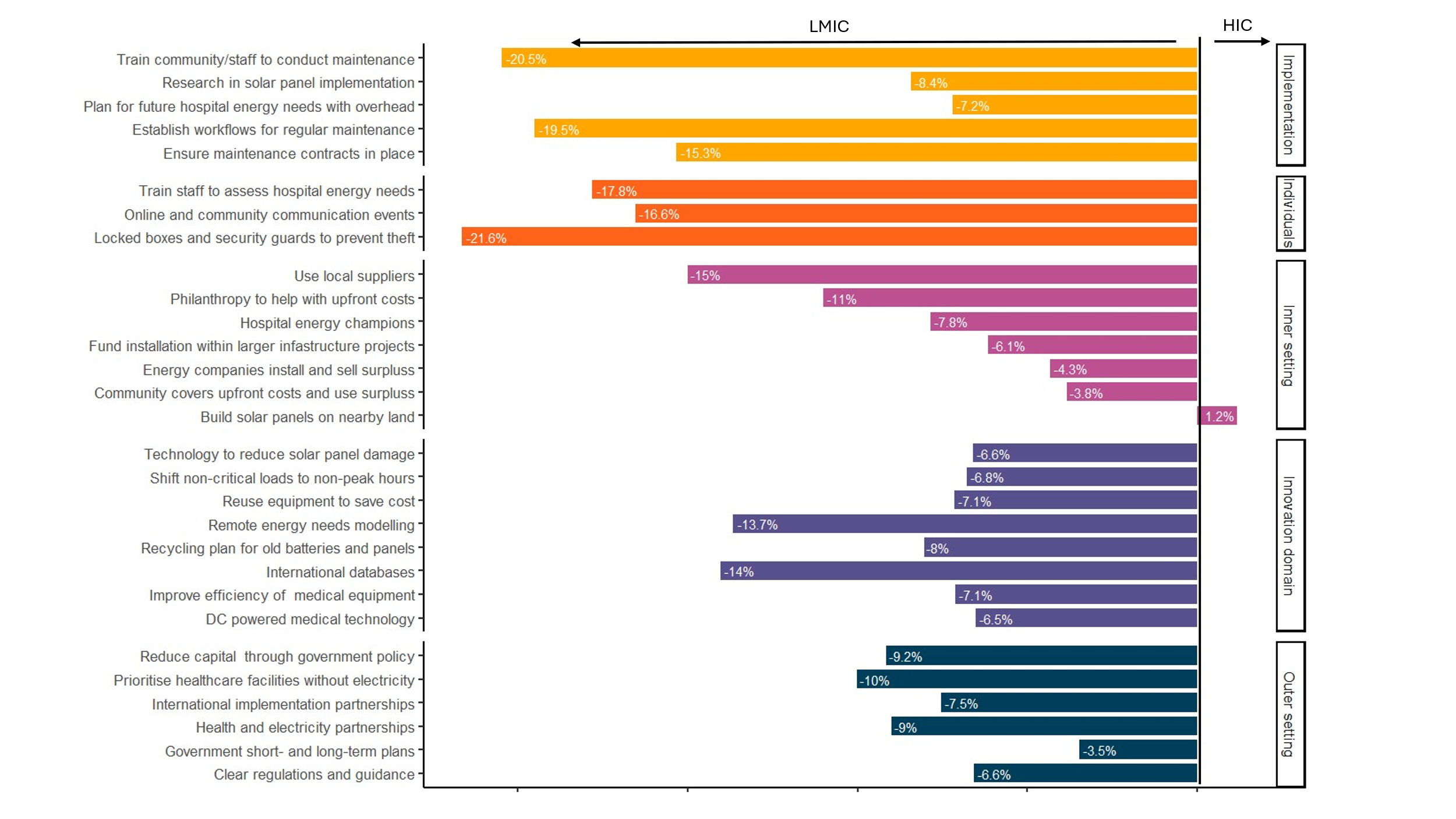


**A)**

**B)**

Supplemental Figure S3: Barriers linked to facilitators linked to Strategy and SOLAR-IT Domain


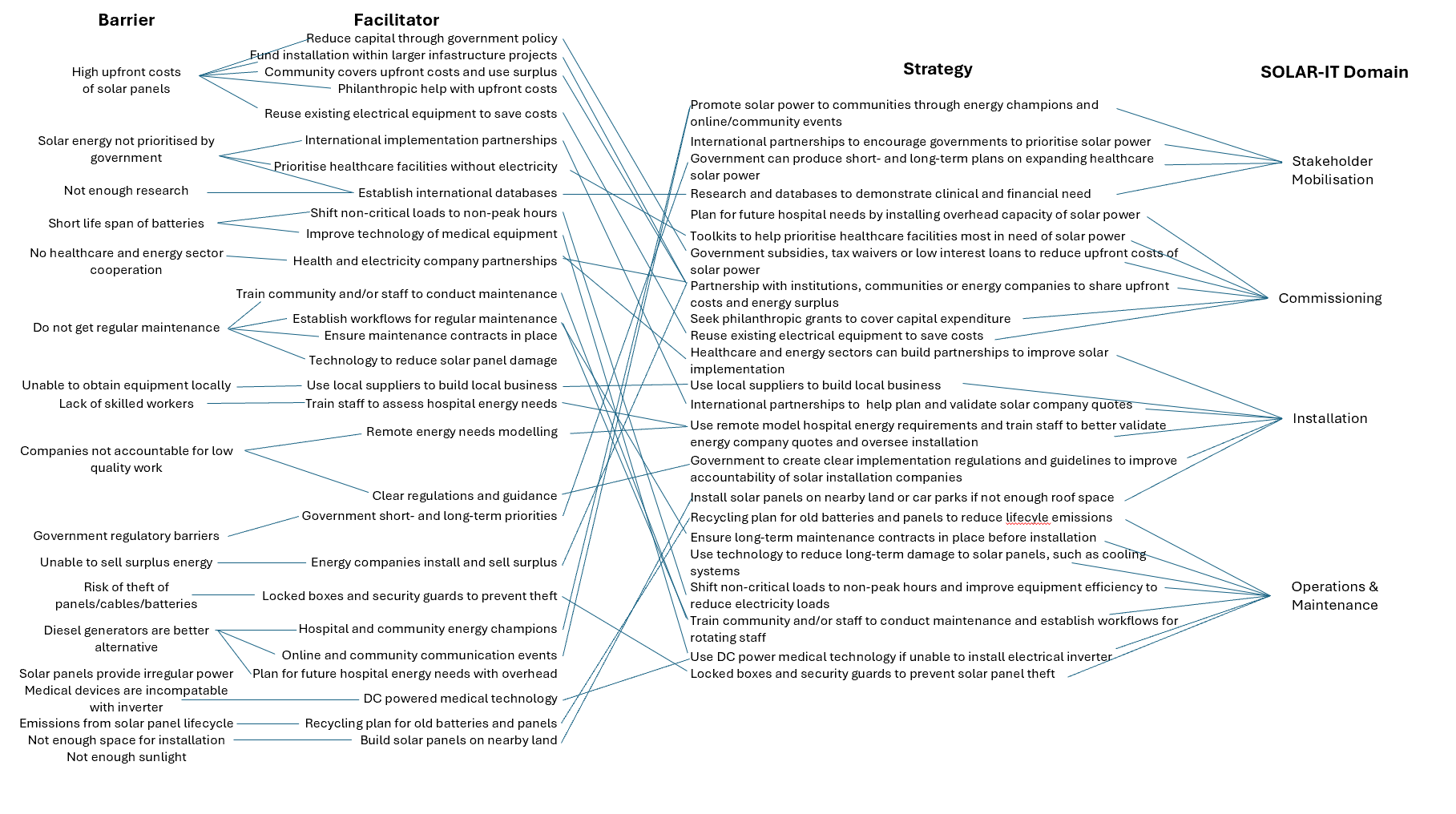

Supplement: online supplemental file 4 [file bmjgh-11-6-s004.docx]
