# Supplementary material for: Barriers, facilitators and strategies for implementing on-site hospital solar power in low- and middle-income countries: a systematic review, global prioritisation survey and development of an implementation tool
Source: BMJ Glob Health. 2026 Jun 18;11(6):e023926. doi: 10.1136/bmjgh-2026-023926 (PMC13288949; doi:10.1136/bmjgh-2026-023926)
Supplement: Supplementary file 5 [file bmjgh-11-6-s005.pdf]

Supplementary S11: Authorship Group

Advisory and writing group

Cortland Linder (UK), Riya Tellis (UK), Adewale Adisa (Nigeria), Dhruva Ghosh (india), Atul Suroy (India), Deepak Singh (India), Nick Marshall (UK), Oliver Penney (UK), Parvez D Haque (India), Virginia Ledda (UK), Sivesh Kamarajah (UK), Liam Phelan (UK), Theophilus Teddy Kojo Anyomih (India), James Glasbey (UK), JC Allen Ingabire (Rwanda), Jane Acquaye (Ghana), Larua Martinez (Mexico), Antonio Ramos-De la Medina (Mexico), Dmitri Nepogodiev (UK), Aneel Bhangu (UK)

Survey collaborators

Afghanistan: Chidiogo Adiukwu (Kabul; French Medical Institute for Mothers and Children (FMIC)); Lorenzo Epis, O O (Kabul; Aliabad Teaching Hospital)

Albania: Saimir Heta, Ilma Robo (Tirana; University Hospital Center Nene Tereza); Dariel Thereska (Tirana; Universitary Hospital Center ' Mother Theresa' Tiranea)

Algeria: Assia Haif (S; Hopital ElBez CCI)

Argentina: René M. Palacios Huatuco (Buenos Aires; Hospital Italiano de Buenos Aires)

Australia: Amelia Davis (Albany; Albany Health Campus); Amanda Dawson (Gosford; Gosford Hospital); Adam Frankel (Brisbane; Princess Alexandra Hospital); Rhea Liang (Southport; Gold Coast University Hospital); Christopher Seng Hong Lim (Canberra; Canberra Hospital); Geraldine Ooi (Melbourne; Monash Medical Center); Maurizio Pacilli (Melbourne; Monash Childrens Hospital); Tarik Sammour (Adelaide; Royal Adelaide Hospital); Jonathan Sivakumat (Melbourne; Western Health - Footscray hospital and Sunshine hospital); Enoch Wong (Melbourne; Box Hill Hospital); Takako Yabe (Wollongong; Wollongong Public Hospital)

Austria: Anja Imsirovic (Innsbruck; Innsbruck Medical University); Robert Pillerstorff (Vienna; General Hospital of Vienna); Thomas Rhomberg (Kagenfurt; Klinikum Klagenfurt am Wörthersee)

Bahrain: Fayza Haider (Manama; Salmaniya Medical Complex)

Bangladesh: John Brigen Mollick, Willem Jan Oosterkamp (Dinajpur; Lamb Hospital)

Belarus: Dmitry Adamovich (Gomel; Gomel State Medical University); Valentin Bereschenko (Gomel; Gomel Regional Specialized Clinical Hospital); Andrey Litvin (Gomel; Gomel University Clinic, Gomel State Medical University)

Belgium: Evelyne Corthouts (Ypres; Jan Yperman); Bert Dhondt (Gent; University Hospital of Ghent)

Bosnia and Herzegovina: Mateo Bevanda (Mostar; SKB University Clinical Hospital Mostar)

Brazil: Fernando Marson (Bragança Paulista; Hospital Universitário São Francisco de Assis na Providência de Deus); Carolina Panis (Francisco Beltrão; Hospital Regional Do Sudoeste Do Paraná)

Bulgaria: Elitsa Gyokova (Pleven; University hospital Saint Marina Pleven); Evguenia Hristova (Sofia; Fifth City Hospital Sofia - 5th MBAL)

Burundi: Jason Fader (Kibuye; Kibuye Hope Hospital)

Canada: Jessica G.y. Luc (Vancouver; Bc); Mahesh Nagappa (London; London Health Sciences Centre and St Josephs Health Care London)

China: Wah Yang (Guangzhou; The First Affiliated Hospital of Jinan University)

Colombia: Camilo Cetares (Bogota; Fundacion Santa Fe de Bogota); Daniel Garcia (Bogota; Central military hospital); Albaro José Nieto Calvache (Cali; Fundación Valle del Lili); Juan Sanjuan (Cartagena; Hospital Serena del Mar)

Croatia: Goran Augustin, Branko Bogdanic (Zagreb; University Hospital Centre Zagreb); Suzana Konjevoda (Zadar; Zadar General Hospital); Ileana Lulic, Jadranka Pavicic Saric (Zagreb; Clinical Hospital Merkur)

Ecuador: Jorge Oliveros-rivero (Guayaquil; Hospital de ni)

Egypt: Ahmed Saber Abdelrahman (Cairo; Giza International Hospital); Mohamed Al Sayed, Ahmed Sabry (Alexandria; Alexandria Main University Hospital); Ahmed Azam, Ali Elhadidy, Selmy S Awad (Mansoura; Mansoura University Hospital); Omnia Eldesouky (Alexandria; Ras El Tin General Hospital); Mahmoud Elfiky (Cairo; Cairo University Hospital); Mohamed El-kassas (Egypt; Helwan University Hospital); Ahmed Maher (Assiut; Assiut University Children Hospital); Mohamed Sherif Ali Ahmed (El Gharbia; El Mahalla General Hospital); Ahmed Tawheed (Cairo; Endemic Medicine Department)

Ethiopia: Rahel Abebayehu Assefa (Addis Ababa; St Peter's Specialised Hospital); Beshea Fufa (Naqamte; Wallaga University Comprehensive Specialized Hospital); Asfaw Kibret (sidama; Leku general hospital); Nathan Suga (Addis Ababa; Myungsung Christian Medical Centre); Adugnaw Worku (Mizan; Mizan_Tepi University Teaching Hospital); Addis Yeshitila (Shewarobit; Yifat Primery Hospital)

Fiji: Maloni Bulanauca (Labasa; LABASA hospital)

Georgia: Zaza Demetrashvili (Tbilisi; N.Kipshidze Central University Clinic)

Germany: Arne Böttcher (Hamburg; University Medical Center Hamburg-Eppendorf); Johannes Doerner, Christoph Mallmann (Wuppertal; Helios Universitätsklinikum Wuppertal (Universität Witten/Herdecke)); Stabsstelle Energiemanagement Gb 6 Facility Management Kaufmännische Direktio, Gregor Massoth (Bonn; University Hospital Bonn); Jan Goedeke (Munich; Ludwig Maximilian University of Munich - Innenstadt); Matthias Janda (Halle; Diakoniekrankenhaus Halle); Christian Konrads (Stralsund; Klinikum Stralsund Hospital); Michael R. Mallmann (Cologne; Cologne-Holweide Medical Center (CHMC)); Ulrich Ronellenfitsch (Halle; University Hospital Halle); Philipp Zehnder (Munich; Klinikum Rechts der Isar TUM School of Medicine); Michael Zyskowski (Munich; TUM University Clinic, Klinikum rechts der Isar)

Ghana: Ralph Armah (Accra; Greater Accra Regional Hospital)

Greece: Pantelis Antonakis (Athens; Aretaieion Hospital); Konstantinos Bouchagier, Elias Liolis (Patras; General University Hospital of Patras); Alexandros Chamzin, Evgenia Mela (Athens; Hippocratio General Hospital); Christos Damaskos (Athens; N.S. Christeas Department Of Surgery); Spiros Delis (Athens; Konstantopouleio Hospital is); Eleni Effraimidou (Alexandroupolis; Alexandroupolis University General Hospital); Nikolaos Garmpis, Michael Spartalis (Athens; Sotiria General Hospital of Thoracic Diseases); Orestis Ioannidis (Thessaloniki; 'G.Papanikolaou' General Hopsital of Thessaloniki); George Kagadis (Rion; University Hospital of Patras); Paraskevi Katsakiori (Akrata; Health Centre of Akrata); Ioannis Katsaros (Athens; Laiko University Hospital); Dimitris Korkolis (Athens; Hellenic Anticancer Hospital of Athens " Saint Savvas"); Andreas Larentzakis (Athens; Athens Medical Center); Stavros Chrysovalantis Liapis, Konstantinos Perivoliotis (Volos; Achillopoyleio General Hospital of Volos); Dimitrios Manatakis (Athens; Naval And Veterans Hospital); Francesk Mulita (Patras; General Hospital Of Eastern Achaia - Unit Of Aigio); Spyros Papadoulas (PATRAS; UNIVERSITY GENERAL HOSPITAL OF PATRAS); Konstantinos Paraskevopoulos (Thessaloniki; George Papanikolaou General Hospital of Thessaloniki); Thalia Petropoulou (Athens; Athens Euroclinic); Konstantinos Roditis, Paraskevi Tsiantoula (Athens; Korgialenio-Benakio Hellenic Red Cross Hospital); George Stavrou (Athens; 417 Army Equity Fund Hospital); Ioannis Triantafyllidis (Kavala; Kavala General Hospital)

Hungary: Attila Zaránd (Budapest; St John's Hospital)

India: Gaurav Agarwal (Lucknow; Sanjay Gandhi Post Graduate Institute Of Medical Sciences); Prafull Kumar Arya (Ludhiana; Satguru Partap Singh Hospital); Lovenish Bains, Lalit Gupta (New Delhi; Maulana Azad Medical College); Somprakas Basu (Rishikesh; All India Institute of Medical Sciences (Aiims)); Prateek Behera, Nitu Mishra (Bhopal; All India Institute of Medical Sciences Bhopal); Mohammad Arfat Ganiyani (Mumbai; Grant Government Medical College and Sir JJ group of hospitals mumbai); Rahul Ghodke (Navi Mumbai; Sanjay Clinic Matoshree Hospital); Krishna Kumar Govindarajan (Pondicherry; Jawaharlal Institute of Postgraduate Medical Education and Research); Amit Gupta (Rishikesh; AIIMS RISHIKESH); Ashish Gupta (Gurugram; Artemis Health Institute); Sachin Kale (Navi Mumbai; D Y Patil Hospital); Madhivanan Karthigeyan, Vishal Kumar (Chandigarh; Postgraduate Institute of Medical Education & Research); Nischal Krishnappa (Bangalore; bgs gims hospital); Shrirang Kulkarni (Kanpur; 7 Air Force Hospital Kanpur); Mrinmoy Kundu (Bhubaneswar; Institute of Medical Sciences and SUM Hospital); Rudrakshi Mahaldar, Saurabh Raj (West Bengal; All India Instititute of Medical Sciences Kalyani); Kashish Malhotra (Hapur; Rama Medical College Hospital and Research Centre); Dattatreya Mukherjee (Raiganj; Raiganj Government Medical College and Hospital); Sathish Muthu (Karur; Government Medical College Hospital); Bijnya Panda, Sujit Tripathy (Bhubaneswar; All India Institute Of Medical Sciences - Bhubaneswar); Piyush Ranjan (New Delhi; All India Institute of Medical Sciences); Manjula S (Mysore; JSS Hospital); Naveen Sharma, Mahendra Singh (Jodhpur; All India Institute of Medical Sciences (AIIMS)); Sunita Singh (Raebareli; All India Institute of Medical Sciences); Vivek Sodhai (Pune; Sancheti Institute for Orthopaedics & Rehabilitation); Hari Kesh Yadav (GORAKHPUR; ALL INDIA INSTITUTE OF MEDICAL SCIENCES)

Indonesia: Christopher Ryalino (Badung; Bali)

Iran: Sayedali Ahmadi (Unknown; Rasool-e-Akram Hospital); Reza Assadi (Mashhad; Imam Reza hospital); Hossein Yusefi (Qom; Imam Reza Hospital)

Iraq: Furqan R. Hassooni (Najaf; Al-Sader Medical City)

Ireland: Michael Kelly (Dublin; St. James's Hospital)

Italy: Michele Ammendola, Giuseppe Curro (Catanzaro; University Hospital 'R. Dulbecco' of Catanzaro); Lorenzo Andreani (Pisa; Azienda Ospedaliero Universitaria Pisana); Michela Angelucci, Gianluca Mascianà, Biagio Zampogna (Rome; Fondazione Policlinico Universitario Campus Bio Medico Of Rome); Amedeo Antonelli, Francesco Giovanardi (Rome; Policlinico Tor Vergata Hospital); Francesca Ascari (Carpi; Ramazzini); Antonio Azzinnaro (Genoa; E.O. Ospedali Galliera); Alberto Balduzzi (Verona; Ospedale Pederzoli); Fabio Barra (Genoa; IRCCS Ospedale Policlinico San Martino); Pietro Bisagni (Lodi; Ospedale Maggiore di Lodi); Giorgio Bogani (Milano; Fondazione IRCCS Istituto Nazionale dei Tumori); Riccardo Borreca (Florence; Careggi university hospital); Giacomo Calini, Diego Raimondo (Bologna; IRCCS Azienda Ospedaliero-Universitaria di Bologna); Tommaso Campagnaro (Verona; Azienda Ospedaliera Universitaria Integrata di Verona); Marianna Capuano, Agostino Fernicola, Giuseppe Palomba (Naples; Federico II University of Naples); Filippo Carannante, Fausto Rosa (Rome; Fondazione Policlinico Universitario Agostino Gemelli); Fabio Carbone (Milan; Istituto Europeo di Oncologia - IRCCS -Milano); Francesco Castagnini, Linda Castagnini, Stefano Lucchini (Bologna; IRCCS Istituto Ortopedico Rizzoli); Pasquale Cianci, Grazisa Scialandrone (Andria; Lorenzo Bonomo); Guglielmo Clarizia (Sondrio; Ospedale di Sondrio (ASST Valtellina e Alto Lario)); Roberto Colasanti (Cesena; Ospedale M. Bufalini); Mario D'oria (Trieste; Cattinara University Hospital); Giuseppe Evola (Catania; ARNAS Garibaldi Hospital); Davide Ferrari (Milan; Fondazione IRCCS Istituto Nazionale dei Tumori); Robert Fruscio (Monza; IRCCS San Gerardo); Marco Giacometti (Guastalla; Civil Hospital - Ausl-Irccs Of Reggio Emilia); Simone Gianazza (Tradate; Lombardy); Francesco Giovinazzo (treviso; San Camillo); Roberta Granata, Massimiliano Veroux (Catania; Azienda Ospedaliero- Universitaria Policlinico San Marco); Giovanni Battista Levi Sandri (Colleferro (Rome); L. P. Delfino District Hospital); Vincenzo Lizzi (Foggia; Ospedali Riuniti Azienda Ospedaliera Universitaria); Elisa Gavina Lutzoni (Sassari; Cliniche San Pietro); Federico Maggi (Rome; Sant'Andrea Hospital); Jacopo Martellucci (Firenze; Azienda Ospedaliera Universitaria Careggi); Gennaro Martines (Bari; Azienda Ospedaliero Universitaria Policlinico); Antonio Meola (Trieste; Azienda Sanitaria Universitaria Giuliano Isontina); Walter Milia (Palermo; ARNAS Civico Hospital); Alba Oliva (Genoa; Ospedale Evangelico Internazionale); Angelo Pascale (Potenza; Azienda Ospedaliera Regionale 'San Carlo'); Federica Pederiva (Varese; Filippo Del Ponte Hospital); Teresa Perra, Alberto Porcu (Sassari; Azienda Ospedaliero Universitaria di Sassari); Umberto Perrone (Genova; IRCCS San Martino Polyclinic Hospital); Giovanni Pesenti (Lecco; ASST di Lecco - P.O di Lecco); Arcangelo Picciariello (Castellana Grotte (Ba); IRCCS 'Saverio de Bellis'); Mauro Podda (Cagliari; Cagliari University Hospital); Ivano Raimondo (Olbia; Mater Olbia Hospital); Stefano Rossi (Rome; Ospedale San Filippo Neri); Diego Sasia (Cuneo; Santa Croce and Carle Hospital); Richard Sassun (Desio; Ospedale di Desio ASST Monza); Cosimo Saviello (Battipaglia (SA); Ospedale Santa Maria della Speranza); Leandro Siragusa (Milan; Humanitas); Marco Sparavigna (Savona; San Paolo); Silvia Maria Tenconi (Varese Lombardy; University of Insubria); Daunia Verdi (Venice; Mirano Hospital)

Japan: Daisuke Hashimoto (Osaka; Kansai Medical University); Akira Kuriyama (Okayama; Kurashiki Central Hospital); Masaki Shiota (Fukuoka; Kyushu University)

Jordan: Mohammad Al-ebbini, Shadi Hamouri (Ar Ramtha; King Abdullah University Hospital/ Jordan University of Science and Technology); Isam Bsisu (Amman; Jordan University Hospital); Almu'atasim Khamees, Raghad Yousef Yassin (Irbid; Princess Basma Hospital)

Kazakhstan: Arailym Abilbayeva (Kegen; Kegen Central Regional Hospital); Ylham Annaorazov (Shymkent; City hospital №1); Ubaidilla Datkhayev, Abay Kusainov, Nurgali Rakhymbayev (Almaty; Scientific Center of Pediatrics and Pediatric Surgery); Dimash Davletov, Anel Ibrayeva (Almaty; JSC 'Central Clinical Hospital'); Ildar Fakhradiyev, Alexandr Tarassyukov (Almaty; City Clinical Hospital No. 4); Timur Fazylov, Madi Tleshev, Dilmurat Zairov (Almaty; Almaty City Emergency Care Hospital); Zhumagali Gabjalilov (Almaty; JSC 'Institute Of Surgery'); Neila Karagezova (Almaty; Inclinic); Mukhtar Kulimbet (Shymkent; Regional clinical hospital of the Turkestan region); Sergey Lee, Darina Menlayakova (Almaty; Karasay Central District Hospital); Zhenis Sergazin (Shymkent; City hospital №2); Лаура Сейдуанова (Almaty; KazNMU)

Kenya: Robert Parker (Bomet; Tenwek Hospital); Mark Siboe (Nairobi; AAR HOSPITAL)

Lebanon: Rania Itani (Beirut; Central Military Hospital)

Libya: Bashir Albakosh (Zliten; Zliten Teaching Hospital); Esraa Albatni (Tripoli; Donia-alfarah); Mohamed Alsori (Tripoli; Tripoli Medical Center/ Tripoli University Hospital); Suhaib Ben Khaled (Nalut; Nalut Central Hospital); Elias Elhoudiri, Mohammed Elshaikhy (Benghazi; Al-jalaa Teaching/Trauma Hospital); Fahed Gareb (Zwara; Zwara Maritime Hospital); Abdurrahman Haddud (Tripoli; Alkhadra Alhadba Hospital); Abdulhamid Shaban (Tripoli; Al-Afia Clinic)

Lithuania: Albertas Dauksa (Kaunas; Lithuanian University of Health Sciences hospital Kaunas Clinics); Zygimantas Dauksa (Kaunas; Lithuanian University of Health Sciences Kaunas Clinical Hospital)

Luxembourg: Alessia Fassari (Luxembourg; Centre Hospitalier De Luxembourg)

Malaysia: Mohamed Shafi Mahboob Ali (Pulau Pinang; Hospital Pulau Pinang / Penang Medical College); Shireen Anne Nah (Kuala Lumpur; University Malaya Medical Centre); Abd Jabar Nazimi (Kuala Lumpur; Hospital Canselor Tuanku Muhriz); Roszalina Ramli (Kuala Lumpur; University Kebangsaan Malaysia Medical Centre); Andee Dzulkarnaen Zakaria (Kelantan; Hospital Universiti Sains Malaysia)

Mexico: Fernando Cordera (Mexico City; ABC Medical Center); María Norma Gómez Herrera (Morelia; Hospital General 'Dr Miguel Silva'); Alberto N. Peón (Pachuca; Hospital Español); Enrique Sanchez-valdivieso (Veracruz City; High Specialty Hospital of Veracruz)

Morocco: Othmane Bouanani (FES; Ibn al khattib hospital); Imad El Azzaoui (Rabat; Hôpital Militaire Mohammed V)

New Zealand: Michael Jen Jie Chu (Christchurch; Christchurch Hospital); Anthony Lin (Wellington; Wellington Regional Hospital)

Nigeria: Lukman Abdur-rahman, Hadijat Olaide Raji (Ilorin; University of Ilorin Teaching Hospital); Auwal Adamu, Abdullahi Musa Kirfi (Bauchi; Abubakar Tafawa Balewa University Teaching Hospital Bauchi); Ademola Adebanjo (Abeokuta; Federal Medical Centre Abeokuta);

Ademola Adeyeye (Ado Ekiti; Afe Babalola University Multi-System Hospital); Adewale Adisa, Chizowa Ezeaku, Akaninyene Ubom (Ile-Ife; Obafemi Awolowo University Teaching Hospitals Complex); Cynthia Agbonrofo, Peter Agbonrofo (Benin City; University of Benin Teaching Hospital); Bolaji Akanni (Abakaliki; Alex Ekwueme Federal University Teaching Hospital Abakiliki 2 (AEFUTHA 2)); Akinola Akinmade (Ado-Ekiti; Afe Babalola University Multi-system Hospital); Bamidele Alegbeleye (Ondo; University of Medical Sciences Teaching Hospital Complex); Lofty-john Anyanwu, Mustapha Ibrahim, Abubakar Bala Muhammad, Shamsuddeen Muhammad, Abubakar Bala Muhammad (Kano; Aminu Kano Teaching Hospital); Mutiu Adebayo Bashiru (Nguru; Federal Medical Centre Nguru); Hannah Chukwu (Enugu; Enugu State University Teaching Hospital); Isaac Chukwu (Umuahia; Federal Medical Centre Umuahia); Ifeanyichukwu Egbuchulem (ibadan; University College Hospital Ibadan); Samuel Ekpemo (umuahia; Federal medical centre); Ndukauba Eleweke (ABA; ABIA STATE UNIVERSITY TEACHING HOSPITAL); Uchechukwu Ezomike (Enugu; university of Nigeria teaching hospital ituku ozalla Enugu); Emmanuel Ezunu (Asaba; Federal Medical Center Asaba); Stephen Gana, Tunde Sholadoye, Musliu Adetola Tolani (Zaria; Ahmadu Bello University Teaching Hospital); Olusola Idowu, Taiwo Lawal, Habeeb Ogundipe, Ajibola Oladiran, Ebere Ugwu, Ifeanyichukwu Ugwu (Ibadan; University College Hospital); Fred Sunday Ige-orhionkpaibima (Asaba; Federal Medical Centre, Asaba); Christian Isichei (Jos; Faith Alive Foundation); Innih Kadiri, Julius Olaogun (Ado-Ekiti; Ekiti State University Teaching Hospital); Jerry Godfrey Makama, Amina Mohammed-durosinlorun (Kaduna; Barau Dikko Teaching Hospital); Tajudeen Mohammed (Ilesa; Obafemi Awolowo University Teaching Hospitals Complex Wesley Guild Hospital Unit); Eghosa Morgan (City; AWESOME GRACE HOSPITAL); Ibukunolu Ogundele (Sagamu; Olabisi Onabanjo University Teaching Hospital); Olakayode Ogundoyin (Ibadan; O.L.A. Catholic Hospital); Olumuyiwa Ogunlaja (ogbomoso; Bowen university Teaching Hospitals); Abiodun Okunlola, Tesleem Orewole (Ido Ekiti; Federal Teaching Hospital); Yusuf Oshodi (Ikeja; Lagos State University Teaching Hospital, Ikeja); Bilal Sulaiman (Abuja; University of Abuja Teaching Hospital); Bashir M Umar (Gombe; Gombe State Specialist Hospital)

North Macedonia: Toni Risteski (Skopje; University Clinic for Pediatric Surgery)

Pakistan: Faaiz Ali Shah (Peshawar; Lady Reading Hospital); Mariyah Anwer (Karachi; Jinnah postgraduate medical center); Usama Kathia (Phalia; THQ Hospital); Dr Sehrish Latif (Islamabad; Shifa international hospital); Waleed Mabood (Peshawar; Mercy Teaching Hospital); Muhammad Usman Malik (Khushab; District Head Quarter); Umar Saeed (Islamabad; International Center Of Medical Sciences Research (Icmsr)); Tazmeen Talia (Hasilpur; Ramay Hospital)

Palestine: Omar Al-haj (Jenin; West Bank); Said Alijla (Gaza; Algerian Especiality Hospital); Mohammed Hajhamad (Nablus; West Bank); Malak Hroub (Jerusalem; West Bank); Sora Nafei (Gaza; Al shifaa hospital)

Panama: Reinaldo Isaacs (Panama; Hospital Regional Rafael Hernandez CSS)

Peru: Cesar Fernando Huaroto Landeo (Lima; Clinica Internacional); Carlos Shiraishi-zapata (Piura; Hospital III José Cayetano Heredia EsSalud Piura); Edilberto Temoche Espinoza (Lima; Hospital Central Luis N. Saenz PNP)

Philippines: Marie Carmela Lapitan (Manila; Philippine General Hospital); Hazel Turingan (Manila; Ospital ng Maynila Medical Center); Cheyene Violanta (Batangas City; Batangas Medical Center)

Poland: Filip Brzeszczynski (Łódź; Copernicus Memorial Hospital); Maciej Matyja (Krakow; Jagiellonian University Medical College); Piotr Stogowski (Gdansk; Szpital im. M. Kopernika / Copernicus Hospital); Leszek Sułkowski (Częstochowa; Regional Specialist Hospital)

Portugal: Nuno Borges (Lisbon; Centro Hospitalar Universitário Lisboa Central); Andre Lazaro (Coimbra; Centro Hospitalar e Universitario de Coimbra)

Qatar: Ayten Saracoglu (Doha; Aisha Bint Hamad Al Attiyah Hospital); Kemal Tolga Saracoglu (Doha; Hazm Mebaireek General Hospital)

Romania: Nicolae-iustin Berevoescu, Draga Mandi (Bucharest; Coltea Clinical Hospital); Elena Chitoran (Bucharest; Institute of Oncology Prof Dr Al Trestioreanu); Ionut Negoi (Bucharest; Emergency Clinical Hospital Bucharest); Andreea Madalina Serban (Bucharest; Maria Sklodowska Curie Emergency Hospital); Elena Tarca (Iasi; Saint Mary Emergency Children Hospital)

Russia: Ruslan Menzulin (Kovrov; First Clinical Medical Center); Ivan Semenenko (Moscow; IM Sechenov First Moscow State Medical University); Mark Tokarev (Moscow; I.M. Sechenov First Moscow State Medical University (Sechenov University))

Saudi Arabia: Nasser Alzerwi (Riyadh; Al Iman General Hospital); Sharfuddin Chowdhury (Riyadh; King Saud Medical City); Arif Hussain (Riyadh; King Abdullaziz Medical City National Guard); Rajkumar Rajendram (Riyadh; King Abdulaziz Medical City)

Serbia: Mihailo Bezmarevic (Belgrade; Military Medical Academy); Ivan Kostić (Čačak; General Hospital Čačak)

Singapore: Han Boon Oh (Singapore; Ng Teng Fong General Hospital); Ming Ngan Aloysius Tan (Singapore; Tan Tock Seng Hospital)

Slovenia: Dejan Bratus (Maribor; University Medical Centre)

Somalia: Mohiaden Jama (Borama; Awdal Hospital); Nur Macawiye Muse (Borama; borama regional hospital)

South Africa: Sebastiano Dos Santos (Delmas; Bernice Samuel Hospital); Zafar Ahmed Khan (Johannesburg; Charlotte Maxeke Johannesburg Academic Hospital); Shrikant Peters (Cape Town; Groote Schuur Hospital)

Spain: María Asunción Acosta-mérida (Las Palmas De Gran Canaria; Hospital Universitario de Gran Canaria Doctor Negrín); Francisco José Almoguera González, Andrea Balla, Lorena Sobrino Brenes (Seville; Hospital Universitario Virgen Macarena); Verónica Alonso (Burgos; Hospital Universitario de Burgos); Martina-aida Angeles, Antonio Gil-moreno, Susana González-suárez (Barcelona; Vall d'Hebron University Hospital); Renan Carlo Colombari, Fernando Fernández Bueno (Madrid; Hospital Central De La Defensa Gomez Ulla); Manuel Diez Alonso (Alcala de Henares. Madrid; Principe de Asturias); Maria Labalde-martinez (Madrid; 12 de octubre university hospital); Manuel López-baamonde, Federico Migliorelli Falcone, Eva Rivas

(Barcelona; Hospital Clinic Barcelona); Lucila Marquez (Madrid; Hospital Central de la Cruz Roja San Jose y Santa Adela); Alberto Martinez De Aragón (Madrid; Hospital Universitario Ramón y Cajal); Javier Ripollés-melchor (Madrid; Infanta Leonor University Hospital); Patrici Serrano Méndez (Madrid; Hospital Clinico San Carlos); Manuel Tousidonis (Madrid; Hospital General Universitario Gregorio Marañón); Marc Vallve-bernal (Tarragona; Hospital Universitari de Tarragona Joan XXIII)

Sri Lanka: Prabath Madhuwantha (Welimada; Base hospital Welimada); Dakshitha Wickramasinghe (Colombo; National Hospital of Sri Lanka)

Sudan: Abdelrahim Abdelrazig Ahmed Ibrahim (Kassala; Kassala teaching hospital); Hajer Alsadeg (Shendi; Aliyaa Almatama); Malaz Azhari (Ed Dueim; Ed Dueim Teaching Hospital); Ibrahim Nagmeldin Hassan (Khartoum; Ibrahim Said rural hospital on the island Aslanj); Nuseiba Osman (Kassala; Police hospital)

Switzerland: Jörn-markus Gass, Julia Mühlhäusser (Luzern; Luzerner Kantonsspital); Johannes Maria Alberto Toti (Bellinzona; EOC Ospedale Regionale di Bellinzona e Valli)

Syria: Ashraf Bakri (Latakia; National Hospital)

Thailand: Suphakarn Techapongsatorn (Bangkok; Vajira hospital)

Tunisia: Montassar Ghalleb, Bouhani Malek (Tunis; Institut Salah Azaiez); Anis Hasnaoui (Menzel Bourguiba; Menzel Bourguiba Hospital); Wiem Rhaiem (sfax; hedi chaker hospital)

Turkey: Omar Alomari (Istanbul; Kartal Dr. Lutfi Kirdar Training and Research Hospital); Yener Aydin, Ali Bilal Ulas (Erzurum; Atatürk University Faculty of Medicine); Seyit Murat Aydın, Muhammed Kerem Deli (Ankara; University Of Health Sciences); Yunushan Furkan Aydoğdu (Ankara; Mamak State Hospital); Ibrahim Buğra Bahadır (Ankara; Sincan Training and Research Hospital); Ahmet Çağlar Bozkurt, Güney Cem Bülbüloğlu (Adana; Moh Adana Kozan State Hospital); Mehmet Çağlar Çakıcı, Arda Isik (Istanbul; Istanbul Medeniyet University); Alp Omer Canturk (Sakarya; Sakarya University Training and Research Hospital); Ali Cantürk, Haluk Kerim Karakullukcu, Nurhilal Kiziltoprak, Omer Faruk Ozkan (Istanbul; Sultan 2. Abdulhamid Han Training and Research Hospital); Bulent Citgez (Istanbul; Memorial Atasehir Hospital); Elif Colak (Samsun; Samsun University); Serap Erel (Ankara; University of Health Sciences Etlik City Hospital); Ergin Erginöz (Istanbul; Istanbul universty - Cerrahpaşa Medical faculty); Özgür Gangal, Yasin Kara (Istanbul; Kanuni Sultan Suleyman Training and Research Hospital); Mert Guler (Istanbul; Gaziosmanpasa Research and Training Hospital); Adnan Gundogdu (Istanbul; Sehit Prof.Dr. İlhan Varank Training and Research Hospital); Erdinc Kamer (izmir; izmir state hospital); Guldeniz Karadeniz Cakmak (Zonguldak; Zonguldak Bulent Ecevit University School of Medicine Research and Training Hospital); Gizem Kiliç Aydoğdu (Ankara; Gazi University school of medicine); Serhat Meric, Banu Yigit (Istanbul; Bagcilar Research And Training Hospital); Cenk Özkan (İstanbul; SBÜ Kanuni Sultan Süleyman EAH); Ahmet Necati Sanli (Gaziantep; Abdulkadir Yuksel State Hospital); Osman Sibic (Mardin; Derik State Hospital); Hanife Seyda Ulgur Genc (Konya; Cihanbeyli State Hospital); Talar Vartanoglu Aktokmakyan (Istanbul; Surp Pirgic Armenian Hospital); Abdullah Hilmi Yilmaz (Van; University of Health Science Van Training and Research Hospital)

Uganda: Bienfait Mumbere (Ishaka-Bushenyi; Kampala International University Teaching Hospital)

Ukraine: Igor A. Kryvoruchko (Kharkiv; Regional Hospital)

United Kingdom: Antonios Athanasiou (Brighton; Royal Sussex county hospital); Avinash Aujayeb (North Shields; Northumbria NHS Hospital Trust); Ajay Belgaumkar (Redhill; East Surrey Hospital); Hannah Beswick (Warwick; South Warwickshire NHS Foundation Trust); George Bethell (Southampton; Southampton General Hospital); Mary Claxton (Taunton; Musgrove Park Hospital); Charis Demetriou (Norwich; Norfolk and Norwich University Hospital); Richard Egan (Swansea; Morriston Hospital Swansea); Dorothy Kufeji (London; Evelina London Children's Hospital); David Mak (Wolverhampton; New Cross Hospital); Ameya Mandrekar (Watford; Watford General Hospital); Mohammed Mohammed (London; Queen Elizabeth Hospital); Sanjay Pandanaboyana (Newcastle Upon Tyne; Newcastle Upon Tyne Hospitals NHS Foundation Trust); Dimitri Pournaras (Bristol; Southmead Hospital); Philip Pucher (Portsmouth; Queen Alexandra Hospital); Ivan Tomasi (London; Guy's and St Thomas' Hospitals); Darmarajah Veeramootoo (Camberley; Frimley Health NHS FT - Frimley Park); Simon Williams (Derby; Royal Derby Hospital); Jared Wohlgemut (Paisley; Royal Alexandra Hospital); Kai Yuen Wong (Cambridge; Addenbrooke's Hospital)

United States: Maleeha Ahmad (Loma Linda; Loma Linda University Medical Center); Mahir Gachabayov (Valhalla; Westchester Medical Center); Nir Horesh (Weston; Cleveland Clinic Florida); Arnav Mahajan, Timothy Weber, Berk Ozmen (Cleveland; Ohio); Dimitrios Moris, Henry Rice (Durham; Nc); Indraneil Mukherjee (New York; Staten Island Universtiy Hosptial - Norhtwell Health); Matthew Naunheim (Boston; Massachusetts Eye and Ear); Nathan Novotny (Royal Oak; Corewell Health Children's Center); Ami Shah (Chicago; Il)

Yemen: Taher Bin Sumait (Hadhamout; Sayoun General Hospital); Hebat Allah Fadhl (Aden; Al-Gumhouria General Hospital)
